# One-, Two-, and Three-Dimensional Self-Assembly of Atomically Precise Metal Nanoclusters

**DOI:** 10.3390/nano10061105

**Published:** 2020-06-03

**Authors:** Ayano Ebina, Sakiat Hossain, Hikaru Horihata, Shuhei Ozaki, Shun Kato, Tokuhisa Kawawaki, Yuichi Negishi

**Affiliations:** 1 Department of Applied Chemistry, Faculty of Science, Tokyo University of Science, Kagurazaka, Shinjuku-ku, Tokyo 162-8601, Japan; ayano0may9@gmail.com (A.E.); hisakiat@gmail.com (S.H.); konpei1082saikou@gmail.com (H.H.); shuheiozaki@icloud.com (S.O.); syunpeeee9@gmail.com (S.K.); kawawaki@rs.tus.ac.jp (T.K.); 2 Research Institute for Science & Technology, Tokyo University of Science, Shinjuku-ku, Tokyo 162-8601, Japan; 3 Photocatalysis International Research Center, Tokyo University of Science, 2641 Yamazaki, Noda, Chiba 278-8510, Japan

**Keywords:** metal cluster, one-dimensional connected structure, two-dimensional connected structure, three-dimensional connected structure, metal–organic framework, photoluminescence, electrical conductivity

## Abstract

Metal nanoclusters (NCs), which consist of several, to about one hundred, metal atoms, have attracted much attention as functional nanomaterials for use in nanotechnology. Because of their fine particle size, metal NCs exhibit physical/chemical properties and functions different from those of the corresponding bulk metal. In recent years, many techniques to precisely synthesize metal NCs have been developed. However, to apply these metal NCs in devices and as next-generation materials, it is necessary to assemble metal NCs to a size that is easy to handle. Recently, multiple techniques have been developed to form one-, two-, and three-dimensional connected structures (CSs) of metal NCs through self-assembly. Further progress of these techniques will promote the development of nanomaterials that take advantage of the characteristics of metal NCs. This review summarizes previous research on the CSs of metal NCs. We hope that this review will allow readers to obtain a general understanding of the formation and functions of CSs and that the obtained knowledge will help to establish clear design guidelines for fabricating new CSs with desired functions in the future.

## 1. Introduction

### 1.1. Metal Nanoclusters for Nanotechnology

Nanotechnology is technology to precisely manufacture small structures. In many countries, nanotechnology is being promoted as a national policy. Progress of nanotechnology allows information and functions to be integrated in smaller spaces, making it possible to manufacture devices with more functions at the same scale. In addition, since nanotechnology makes it possible to integrate the same function in a smaller volume than is the case for current devices, it is expected that devices will be downsized, which will increase their portability. This eliminates the need for the user to be dependent on the device location, which could solve the problems such as crowding and traffic jams and allow users to manage their time more effectively. In addition, the progress of nanotechnology has many advantages, such as saving resources and energy and decreasing waste and environmental damage [1].

**Figure 1:**
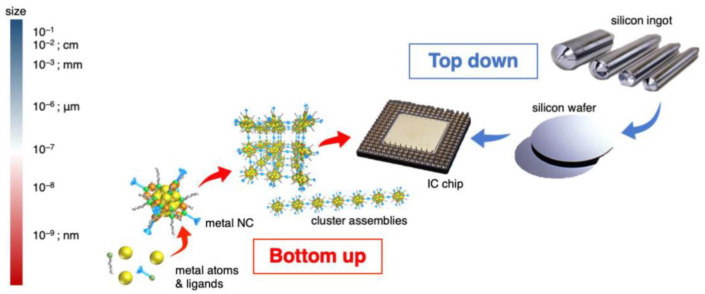
Fine processing techniques, including top-down (cyan) and bottom-up (red) methods. IC = integrated circuit.

Techniques to fabricate small materials can be roughly divided into two categories. One category is top-down methods, in which a desired structure is produced by decreasing the size of a larger substrate (Figure 1). Nanotechnology has been supported by the development of top-down methods. For example, increased functionality and miniaturization of electronic devices have been realized by the progress of top-down techniques. However, when a fine structure is manufactured by using tools (light, electron beam, scanning probe microscope, etc.), it is difficult to manufacture nanostructures with finer accuracy than that of the tools. Therefore, in recent years, bottom-up methods that assemble nanostructures from atoms and molecules have attracted attention (Figure 1) [1].

Metal nanoclusters (NCs), which consist of several, up to about one hundred, metal atoms [1,2,3,4,5,6,7,8,9,10], are nanomaterials that can be synthesized by bottom-up methods. Metal NCs are not only small (<2 nm in size), but they also show physical/chemical properties and functions which are different from those of the corresponding bulk metals [11,12,13,14,15,16,17,18,19,20,21,22,23,24,25,26,27,28,29,30,31,32,33,34,35]. Furthermore, the physical/chemical properties and functions of metal NCs change considerably depending on the number of constituent atoms [36,37,38,39,40,41,42,43,44,45,46,47,48,49,50,51,52,53,54,55,56,57,58,59,60,61,62,63,64,65]. Therefore, if the number of constituent atoms of metal NCs is controlled, it is possible to produce various physical/chemical properties and functions by using only one type of metal element. If several types of elements can be used, it becomes possible to obtain more functionalities [66,67,68,69,70,71,72,73,74,75,76,77,78,79,80,81,82]. For these reasons, metal NCs have been attracting considerable attention as a central material in nanotechnology. In recent years, it has become possible to synthesize such metal NCs precisely at the atomic and molecular level by using thiolate (SR) [2,66], alkyne [59,83], phosphine [9,84,85,86,87,88,89,90,91,92], carbon monoxide [93,94,95,96,97,98,99,100], and dendrimers [7] as protective organic molecules. Investigation of the obtained precise metal NCs has revealed their geometrical structure (aggregation pattern of metal atoms) and the influences of miniaturization [1,2,3,4,5,6,7,8,9,10,11,12,13,14,15,16,17,18,19,20,21,22,23,24,25,26,27,28,29,30,31,32,33,34,35,36,37,38,39,40,41,42,43,44,45,46,47,48,49,50,51,52,53,54,55,56,57,58,59,60,61,62,63,64,65] and alloying [66,67,68,69,70,71,72,73,74,75,76,77,78,79,80,81,82] on the electronic structures and physical/chemical properties of metal NCs. In parallel, research on the applications of the optical properties and catalytic activity of metal NCs is being actively conducted [81,101,102,103,104,105,106,107,108].

### 1.2. Controlled Assembly of Metal Nanoclusters

As mentioned above, metal NCs show promise as constituent units of functional nanomaterials. Gold (Au) NCs have already been put to practical use in the fields of sensors, catalysts, and paints. On the other hand, at present, electronic devices are manufactured by top-down methods. Therefore, to replace the components of current devices with metal NCs, it is necessary to grow metal NCs to a size that allows their combination with structures manufactured by top-down methods. Moreover, in other applications, the small size of metal NCs often makes them difficult to handle. To realize nanodevices and next-generation materials with the advantageous characteristics of metal NCs, it is essential to establish techniques to assemble metal NCs to a size that makes them easy to handle.

To form a one-dimensional (1D) arrangement of metal NCs, templates [109,110] and host–guest interactions [111] are extremely effective. Two-dimensional (2D) and three-dimensional (3D) arrays of metal NCs can be fabricated by Langmuir–Blodgett [112] and alternate adsorption methods. There are many reports in which metal NCs are arranged in one, two, and three dimensions by using these methods. However, in the structures produced by these methods, the metal NCs are not regularly arranged in a strict sense. Since the conductivity (σ) of metal NCs changes exponentially with the length of the insulating organic ligands [113], the existence of a distribution in the distances between NCs (i.e., the length of the insulating part) is undesirable when applying the assembled metal NCs in electronic devices. Furthermore, although the Langmuir–Blodgett and alternate adsorption methods are suitable for arranging metal NCs in a wide size area, they are not suitable for arranging those in a tiny area.

**Figure 2:**
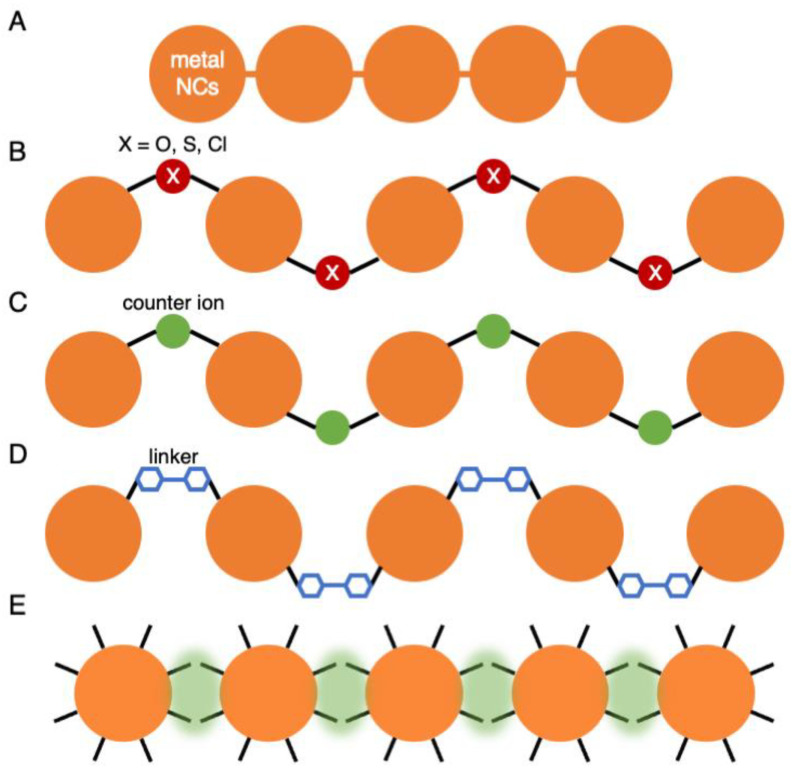
Representative methods for connecting metal NCs: (**A**) formation of metal−metal bond; (**B**) formation of Ag−O, Ag−S, and Ag−Cl bonds; (**C**) control of counterions; (**D**) introduction of linker molecules; and (**E**) use of inter-ligand interactions. In this review, 1D, 2D, and 3D CSs formed by inter-ligand interactions (**E**) are not introduced.

Metal NCs are regularly arranged in single crystals. Therefore, it is possible to produce precise structures in which metal NCs are regularly connected in one, two, and three dimensions by crystallizing them while including an ingenious means to connect metal NCs. In fact, many crystals in which metal NCs are regularly linked by such a method have been reported in recent years. These structures contain strong bonds, such as Au−Au, Au−silver (Ag), Ag−oxygen (O), Ag−sulfur (S), Ag−chloride (Cl), Ag−nitrogen (N), cesium (Cs)−S, or hydrogen (H) bonds [114], and weak interactions, such as π−π, anion−π, cation···π, aryl CH···Cl, and van der Waals interactions [115] (Figure 2). Similar to supramolecules, molecular assemblies [116], and metal–organic frameworks (MOFs) [117] that are self-assembled from metal ions and organic molecules, connected structures (CSs) of metal NCs can be formed by self-assembly during crystallization, using these bonds and interactions. Such structures, which could also be called “suprametal NC crystals”, exhibit physical/chemical properties different from those of an individual metal NC. Thus, the formation of CSs not only increases the size of the structure, but also enables the application of NCs in new fields.

### 1.3. Contents of This Review

In recent years, it has become possible to control not only the geometrical structure of metal NCs, but also 1D, 2D, and 3D CSs of metal NCs. Further development of these techniques will lead to novel nanomaterials possessing the characteristics of metal NCs. Such development may enable a future in which metal NCs are applied in devices. However, since these studies have been initiated in recent years, there have been few review articles focusing on 1D, 2D, and 3D CSs of metal NCs [118,119]. 

In this review, we summarize the existing research, with the purposes of understanding the current situation regarding these structures and giving perspective regarding clear design for producing new 1D, 2D, and 3D CSs with desired functions.

This review is structured as follows. Section 2 outlines the fabrication of 1D CSs consisting of metal NCs, their geometrical structures, and physical/chemical properties. Then, Section 3 and Section 4 present research on 2D and 3D CSs, respectively. After summarizing this review article in Section 5, a brief future outlook is described in Section 6.

It should be noted that 1D, 2D, and 3D CSs of metal NCs can be formed by methods other than crystallization [120,121]; for example, hydrophilic Au NCs have been arranged in 1D and 3D form by the Xie’s group, although these NCs have not been crystallized. However, in this review, only the CSs of metal NCs in crystals are summarized, because our focus is on the regularly CSs in a strict sense. In addition, we described the synthesis methods only for the several examples. Thus, we recommend the readers who want to know the detail of synthesis methods for each example to refer to each original paper.

## 2. One-Dimensional Structures

**Scheme 1:**
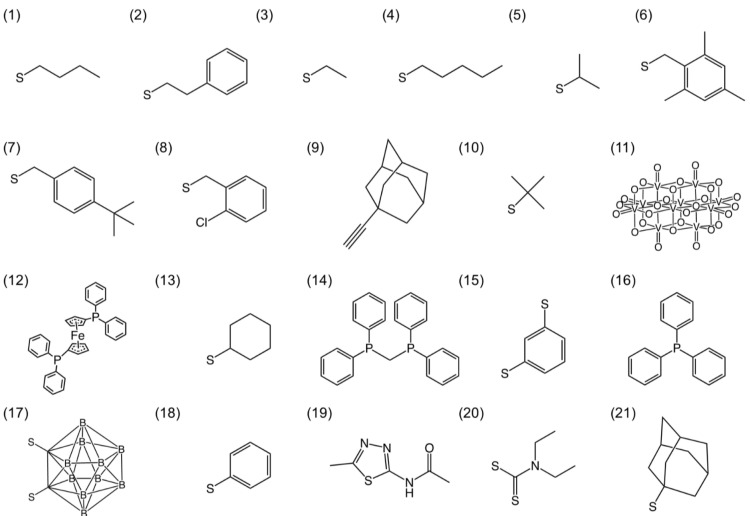
Molecules used in the synthesis of metal NCs: (**1**) S-Bu, (**2**) PET, (**3**) S-Et, (**4**) S-Pen, (**5**) S-*i*Pr, (**6**) SCH_2_Ph(CH_3_)_3_, (**7**) SCH_2_Ph*^t^*Bu, (**8**) SCH_2_PhCl, (**9**) A-Adm, (**10**) S-*^t^*Bu, (**11**) V_10_O_28_^6−^, (**12**) dppf, (**13**) S-*c*-C_6_H_11_, (**14**) DPPM, (**15**) BDT, (**16**) PPh_3_, (**17**) DT-*o*-C, (**18**) S-Ph, (**19**) N-L, (**20**) C_5_NS_2_H_10_, and (**21**) S-Adm.

**Scheme 2:**
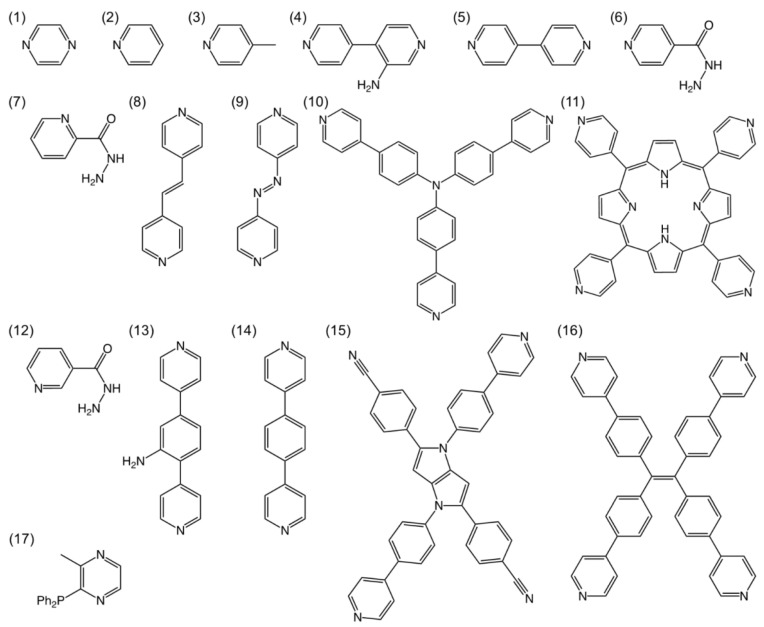
Linker molecules used to connect metal NCs: (**1**) pyrazine, (**2**) pyridine, (**3**) *p*-methylpyridine, (**4**) bpy-NH_2_, (**5**) bpy, (**6**) *p*-iah, (**7**) *o*-iah, (**8**) bpe, (**9**) dipyridin-4-yl-diazene, (**10**) TPPA, (**11**) TPyP, (**12**) *m*-iah, (**13**) bpz-NH_2_, (**14**) 1,4-bis(4-pyridyl)benzene, (**15**) CPPP, (**16**) tppe, and (**17**) mdppz.

The formation of 1D CSs composed of precise metal NCs is important from the viewpoint of the fabrication of controlled nanodevices by bottom-up methods. In this section, we introduce some typical examples of the construction of 1D CSs by the formation of metal−metal bonds (Figure 2A), formation of Ag−O bonds (Figure 2B), control of counterions (Figure 2C), and introduction of linker molecules (Figure 2D). The connection methods, NCs, linkers, year reported, and reference numbers of 1D CSs are summarized in Table 1. Chemical structures of some of the ligands used in these studies are shown in Scheme 1. The chemical structures of organic molecules used as linkers are illustrated in Scheme 2.

### 2.1. Direct Connection via Metal−Metal Bonds

In 2014, Maran et al. [122] fabricated a 1D CS composed of an SR-protected Au 25-atom NC ([Au_25_(SR)_18_]^0^). Since Au_25_(SR)_18_ NCs exhibit high stability among Au*_n_*(SR)*_m_* NCs, their geometrical/electronic structures and physical/chemical properties have been studied extensively [18,27,123,124,125,126,127,128,129,130,131,132,133,134,135,136,137,138,139]. However, because most of the studies were conducted on Au_25_(SR)_18_ in solution and there had been few studies on Au_25_(SR)_18_ in the solid phase, Maran’s group studied the behavior of Au_25_(SR)_18_ in the solid state.

**Figure 3:**
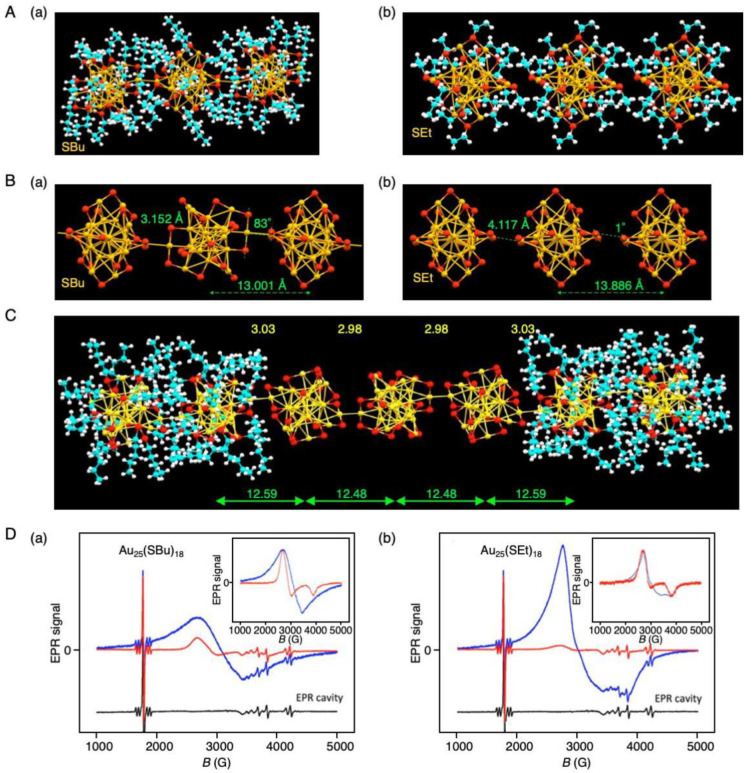
(**A**,**B**) Crystal structures of (a) [Au_25_(S-Bu)_18_]^0^ and (b) [Au_25_(S-Et)_18_]^0^. In (**B**), R groups are omitted for clarity. (**C**) Crystal structure of [Au_25_(S-Pen)_18_]^0^. In (A−C), Au = yellow, S = red, C = light blue, and H = white. (**D**) Comparison of the continuous wave-electron paramagnetic resonance (EPR) spectra of solid (blue traces) and frozen toluene solution (red traces) for (a) [Au_25_(S-Bu)_18_]^0^ and (b) [Au_25_(S-Et)_18_]^0^ at −253 °C. The inset shows the same spectra with normalized peak intensity. The black curve corresponds to the EPR cavity signal, which is subtracted in the inset for clarity. All spectra were obtained by using the following parameters: microwave frequency = 9.733 GHz; microwave power = 150 μW; amplitude modulation = 1 G. Reproduced with permission from References [122,140]. Copyright 2014 American Chemical Society and 2017 American Chemical Society.

In their study, butanethiolate (S-Bu, Scheme 1(1)) was used as the SR ligand. First, [Au_25_(S-Bu)_18_]^−^ anion was synthesized by reducing the Au(I)- S-Bu complex by using sodium borohydride (NaBH_4_). Then, [Au_25_(S-Bu)_18_]^−^ anion was oxidized into neutral [Au_25_(S-Bu)_18_]^0^ in open column packed with silica gel, and single crystals were grown by slow evaporation. Figure 3A(a) shows the geometrical structure of [Au_25_(S-Bu)_18_]^0^ obtained by single-crystal X-ray diffraction (SC-XRD). Each [Au_25_(S-Bu)_18_]^0^ has almost the same framework structure as that of [Au_25_(SR)_18_]^0^ protected by other SR ligands; e.g., phenylethanethiolate (PET, Scheme 1(2)) and ethanethiolate (S-Et, Scheme 1(3)) (Figure 3A(b)) [27,53]. However, Au−Au bonds formed between adjacent NCs in the [Au_25_(S-Bu)_18_]^0^ crystal, unlike the case for other [Au_25_(SR)_18_]^0^ crystals (Figure 3A(a),B(a)). This indicates that [Au_25_(S-Bu)_18_]^0^ is a suitable structural unit to form 1D CSs. The Au−Au distance between adjacent NCs of 3.15 Å was within the range of aurophilic interactions (2.9−3.5 Å) and shorter than the non-bonding Au−Au distance (3.80 Å) estimated from the van der Waals radius of Au. This result indicates that a 1D CS was formed in the crystal structure of [Au_25_(S-Bu)_18_]^0^ via Au−Au bonds. To form such a 1D CS, it was considered that the repulsion between the ligands was suppressed and an attractive force between the ligands was induced because the adjacent NCs twisted and approached each other (twist-and-lock mechanism). It was suggested that 1D CSs did not form when S-Et and PET were used (Figure 3A(b),B(b)) because S-Et has a short alkyl group that leads to a weak attractive force between ligands and PET with a bulky functional group has large steric repulsion between ligands. In 2017, these researchers also succeeded in forming a 1D CS of [Au_25_(S-Pen)_18_]^0^ (S-Pen = pentanethiolate, Scheme 1(4)) [140]. In the same paper, they reported that the distance between NCs was shorter in this 1D CS than in the 1D CS of [Au_25_(S-Bu)_18_]^0^ (Figure 3C). In addition, in 2019, they formed 1D CSs of [Au_24_Hg(S-Bu)_18_]^0^ (Hg = mercury) and [Au_24_Cd(S-Bu)_18_]^0^ (Cd = cadmium), in which one Au of [Au_25_(S-Bu)_18_]^0^ was replaced with Hg or Cd [141].

The same group also revealed that the 1D CSs had electronic structures and physical properties different from those of individual NCs. Since [Au_25_(S-Bu)_18_]^0^ has unpaired electrons, it exhibits paramagnetism in solution. Conversely, the 1D CS of [Au_25_(S-Bu)_18_]^0^ was non-magnetic (Figure 3D) [122]. This change was mainly ascribed to the formation of the 1D CS, which led to the close proximity of NCs, allowing the unpaired electrons of adjacent NCs to form electron pairs. Because of the formation of such electron pairs, the conduction band of the 1D CS was full and its valence band was empty, so the obtained 1D CS was predicted to have the properties of a semiconductor [122].

**Figure 4:**
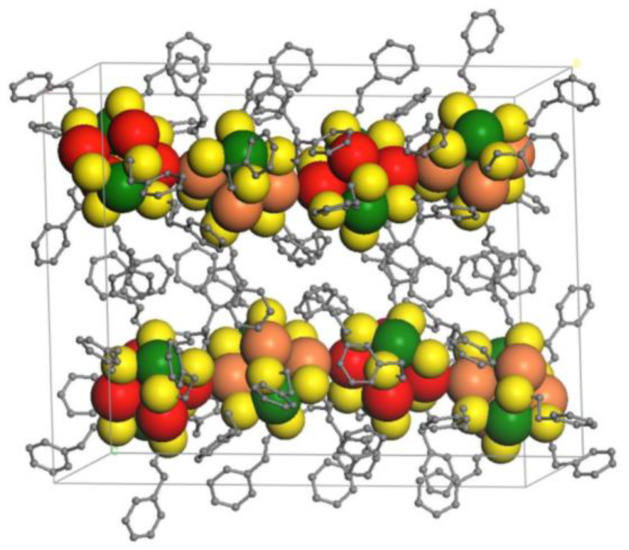
Crystal unit cell of [Au_4_Pd_2_(PET)_8_]^0^. S = yellow, Au = red and orange, Pd = olive, C = gray. Reproduced with permission from Reference [143]. Copyright 2017 Wiley-VCH.

In 2020, we [142] conducted a detailed study on the factors responsible for the formation of 1D CSs via Au−Au bonds by using [Au_4_Pt_2_(SR)_8_]^0^ (Pt = platinum) as the NC. A similar NC, [Au_4_Pd_2_(PET)_8_]^0^ (Pd = palladium), was reported by Wu and colleagues in 2017 (Figure 4) [143]. Although it was not mentioned in their paper, [Au_4_Pd_2_(PET)_8_]^0^ formed a 1D CS in its crystal (Figure 4). Because this type of metal NC has a smaller metal core than that of [Au_25_(SR)_18_]^0^ described above (Figure 4), the distribution of the ligands in this type of NC should change depending on the ligand structure. Moreover, Au and Pt form a stronger bond than that between Au and Pd [144]. Therefore, it was expected that changing Pd to Pt would increase the stability of the NC [145], thereby expanding the variety of ligand functional group structures that can be used in 1D CSs. For these reasons, we chose [Au_4_Pt_2_(SR)_8_]^0^ as the building block of their 1D CS. The SR ligands shown in Scheme 1(2),(5)–(8) were used. Because the functional group structures of these SR ligands differ greatly, it was expected that there would be different ligand–ligand interactions between the resulting NCs.

**Figure 5:**
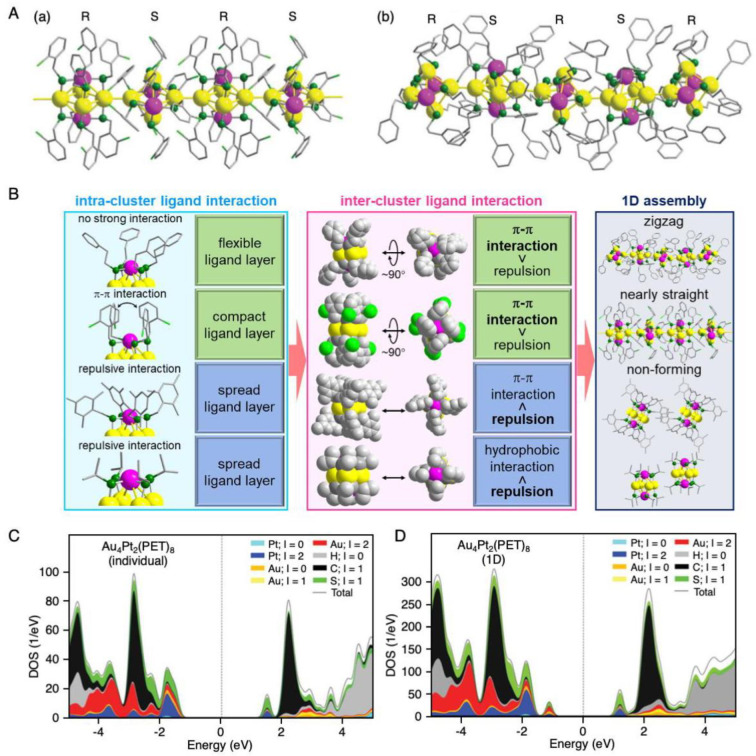
(**A**) Crystal unit cells of (a) [Au_4_Pt_2_(SCH_2_PhCl)_8_]^0^ and (b) [Au_4_Pt_2_(PET)_8_]^0^. Au = yellow, Pt = magenta, S = green, Cl = light green, C = gray. R and S indicate two enantiomers in each NC. (**B**) Relationships between intra-cluster ligand interactions, which are related to the distribution of the ligands within each cluster, inter-cluster ligand interactions, and 1D assembly. Projected density of states of (**C**) an individual [Au_4_Pt_2_(PET)_8_]^0^ NC and (**D**) the 1D CS of [Au_4_Pt_2_(PET)_8_]^0^. Reproduced with permission from Reference [142]. Copyright 2020 Royal Society of Chemistry.

In the experiment, [Au_4_Pt_2_(SR)_8_]^0^ NCs with different SRs were precisely synthesized by reducing the metal−SR complex with NaBH_4_. Each [Au_4_Pt_2_(SR)_8_]^0^ NC was separated from by-products, using open column chromatography, and then single crystals were grown by vapor diffusion. The SC-XRD of the series of [Au_4_Pt_2_(SR)_8_]^0^ crystals revealed the following three points for [Au_4_Pt_2_(SR)_8_]^0^: (1) [Au_4_Pt_2_(SR)_8_]^0^ is a metal NC that can become a structural unit of 1D CSs via Au−Au bond formation (Figure 5A); (2) although all [Au_4_Pt_2_(SR)_8_]^0^ NCs have similar structures, the intra-cluster ligand interactions vary depending on the ligand structure. As a result, the distribution of the ligands in [Au_4_Pt_2_(SR)_8_]^0^ changes depending on the ligand structure; (3) the differences in the ligand distributions influence the inter-cluster ligand interactions, which in turn affect the formation of 1D CSs and change their structure (Figure 5B). These results demonstrate that we need to design intra-cluster ligand interactions, to produce 1D CSs with desired configurations. This study also explored the effects of 1D CS formation on the electronic structure of NCs. The results revealed that the formation of the 1D CS caused the band gap of the NCs to decrease (Figure 5C,D) [142].

**Figure 6:**
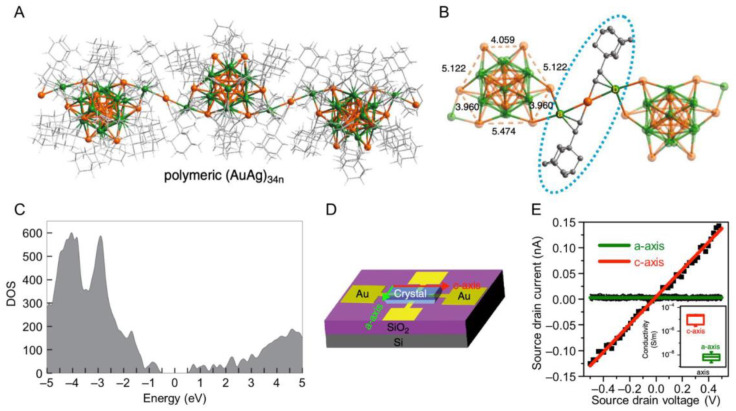
(**A**) Structures of the cluster polymer (approximately orthogonal to the *c*-axis). (**B**) Au–Au distances in the distorted Au_6_ hexagon and Ag–Ag distance in the “Ag–Au–Ag” unit of between alloy NCs. Au/Ag = golden and green, C = gray. All hydrogen atoms are omitted for clarity. (**C**) DFT-computed electronic density of states (DOS) of the cluster polymer crystal. Cluster model was used to build the periodic crystal, and the integration over the Brilloin zone was done in a 4 × 4 × 4 Monkhorst–Pack *k*-point mesh. The band gap is centered around zero. (**D**,**E**) Electrical transport properties of the cluster polymer crystals; (**D**) structure of the polymer crystal FET; (**E**) *I*–*V* plot of the polymer crystal along *a*-axis and *c*-axis, respectively, with the range of corresponding conductivity values shown in the inset. Reproduced with permission from Reference [146]. Copyright 2020 Springer-Nature.

Zhang et al. also very recently reported the formation of 1D CS consisting of the (AuAg)_34_(A-Adm)_20_ alloy NCs (A-Adm = 1-ethynyladamantane; Scheme 1(9)) [146]. For (AuAg)_34_(A-Adm)_20_ alloy NCs, either monomeric NC or 1D CSs were formed depending on the solvent. Monomeric NC could be converted to 1D CSs by dissolving in an appropriate solvent. In the 1D CS, NCs were connected each other via Ag−Au−Ag bond (Figure 6A,B). They studied the electronic structure of the obtained 1D CS by density functional theory (DFT) calculations, which predicted that the single crystals of 1D CS have a band gap of about 1.3 eV (Figure 6C). Field-effect transistors (FETs) fabricated with single crystals of 1D CS (Figure 6D) showed highly anisotropic *p*-type semiconductor properties with ~1800-fold conductivity in the direction of the polymer as compared to cross directions (Figure 6E), hole mobility of ≈0.02 cm^2^/Vs, and an ON/OFF ratio up to ~4000. They noted that the conductivity (1.49 × 10^–5^ S/m) of these crystals in the *c*-crystallographic axis is one-to-three orders of magnitude higher than the values reported for 1D CS consisting of Au_21_ clusters, where 1D CS was formed by modulating the weak interactions in the ligand layers (see Section 2.3). It was interpreted that the conductivity and charge carrier mobility was increased by several orders of magnitude in their 1D CS via direct linking of the metal NCs by the –Ag–Au–Ag– chains in the crystal. They described in this paper that this result holds promise for further design of functional cluster-based materials with highly anisotropic semiconducting properties.

### 2.2. Connection via Ag−O Bonds

**Figure 7:**
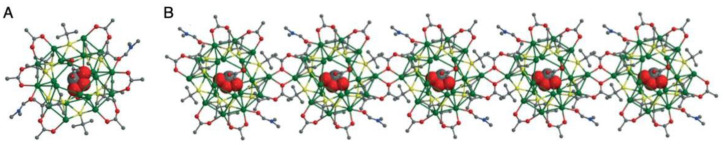
(**A**) Structure of Ag_20_(CO_3_)(S-*^t^*Bu)_10_(CH_3_COO)_8_(DMF)_2_. (**B**) Ball-and-stick view of the 1D chain of Ag_20_(CO_3_)(S-*^t^*Bu)_10_(CH_3_COO)_8_(DMF)_2_. Ag = green, S = yellow, N = blue, O = red, C = gray. Reproduced with permission from Reference [147]. Copyright 2014 Royal Society of Chemistry.

When Ag NCs contain acetic acid ions (CH_3_COO^−^), trifluoroacetic acid ions (CF_3_COO^−^), or nitrate ions (NO_3_^−^) in the ligand layer, it is possible to connect Ag NCs by forming Ag−O bonds. Su et al. [147] first reported the formation of such a 1D CS in 2014. In this study, the 1D CS was obtained by crystallization of the product which was obtained by the reaction between AgS-*^t^*Bu, (NH_4_)_3_[CrMo_6_O_24_H_6_] (Cr = chromium, Mo = molybdenum), Ni(CH_3_COO)_2_ (Ni = nickel), AgCF_3_COO, and AgBF_4_. The each Ag NC had a chemical composition of Ag_20_(CO_3_)(S-*^t^*Bu)_10_(CH_3_COO)_8_(DMF)_2_ (CO_3_^2−^ = carbonate anion; S-*^t^*Bu = *tert*-butylthiolate, Scheme 1(10), DMF = *N,N-*dimethylformamide). The Ag_20_(CO_3_) core of the NC was formed by the aggregation of Ag around CO_3_^2−^ (Figure 7A) as an anion template. In the crystal, the Ag NCs were connected in one dimension via two Ag−O−Ag bonds (Figure 7B). The obtained 1D CS was stable in both solid and solution states, had a bandgap of 3.22 eV, and exhibited reversible thermochromic emission.

**Figure 8:**
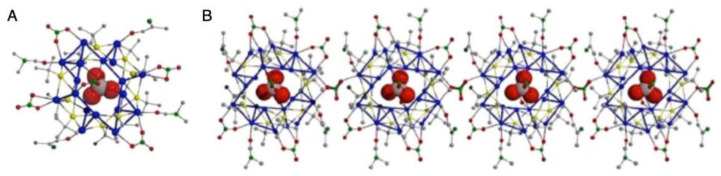
(**A**) Structure of Ag_18_(CO_3_)(S-*^t^*Bu)_10_(NO_3_)_6_(DMF)_4_. (**B**) Ball-and-stick view of the 1D chain of Ag_18_(CO_3_)(S-*^t^*Bu)_10_(NO_3_)_6_(DMF)_4_. Ag = blue, S = yellow, O = red, C = gray, N = green. Reproduced with permission from Reference [148]. Copyright 2017 Wiley-VCH.

Formation of 1D CSs based on a similar principle was also reported by Mak and co-workers in 2017 [148]. In their report, Ag_18_(CO_3_)(S-*^t^*Bu)_10_(NO_3_)_6_(DMF)_4_ was linked by the formation of Ag−O bonds (Figure 8). The Ag_18_(CO_3_) core contained CO_3_^2−^ as an anion template at the center, like Ag_20_(CO_3_). As described in Section 3.1, this group also succeeded in forming a 2D CS of Ag_20_(CO_3_) by changing the SR structure.

**Figure 9:**
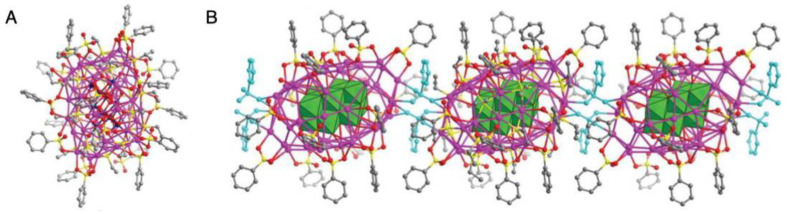
(**A**) Structure of Ag_44_(V_10_O_28_)(S-Et)_20_(PhSO_3_)_18_(H_2_O)_2_ (**B**) 1D chain structure of Ag_44_(V_10_O_28_)(S-Et)_20_(PhSO_3_)_18_(H_2_O)_2_ with all bridging PhSO_3_^−^ ligands highlighted in cyan and V_10_O_28_^6^^−^ anions shown as green polyhedra. Ag = purple, V = dark blue, S = yellow, C = gray, O = red. All H atoms are omitted. Reproduced with permission from Reference [149]. Copyright 2019 Royal Society of Chemistry.

Recently, Sun et al. [149] succeeded in the connection of Ag_44_(V_10_O_28_)(S-Et)_20_(PhSO_3_)_18_(H_2_O)_2_ (V_10_O_28_^6−^, Scheme 1(11), PhSO_3_^−^ = benzenesulfonic acid ion). In this 1D CS, the Ag_44_(V_10_O_28_) core was formed by using the polyoxometalate (POM) V_10_O_28_^6−^ as an anion template (Figure 9A). This was the first report of the formation of a structure in which V_10_O_28_^6−^ was covered with an Ag NC with SR as a ligand. This 1D CS assembled because two Ag−O bonds were formed between two PhSO_3_^−^ in the ligand layer and one Ag_44_(V_10_O_28_)(S-Et)_20_ NC (Figure 9B).

**Figure 10:**
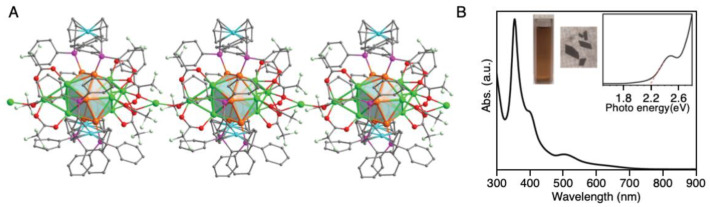
(**A**) View of the whole structure of [Au_7_Ag_9_(dppf)_3_(CF_3_COO)_7_BF_4_]*_n_* (anions and H atoms are omitted for clarity). Ag = green, Au = orange, O = red, P = purple, C = gray, Fe = blue. (**B**) Absorption spectrum of [Au_7_Ag_9_(dppf)_3_(CF_3_COO)_7_BF_4_]*_n_* in CH_2_Cl_2_ solution. Inset: absorption spectrum on the energy scale (eV) and photographs showing actual colors of [Au_7_Ag_9_(dppf)_3_(CF_3_COO)_7_BF_4_]*_n_* in CH_2_Cl_2_ and the crystalline state. Reproduced with permission from Reference [150]. Copyright 2019 Royal Society of Chemistry.

In the 1D CS of [Au_7_Ag_9_(dppf)_3_(CF_3_COO)_7_BF_4_]*_n_* (dppf = 1,1′-bis(diphenylphosphino)ferrocene, Scheme 1(12), BF_4_ = tetrafluoroboric acid) reported by Wang et al. [150] in 2019, each NC was also connected via an Ag−O bond (Figure 10A), although this was not direct connection of metal NCs. In the above three NCs (i.e., Ag_20_(CO_3_)(S-*^t^*Bu)_10_(CH_3_COO)_8_(DMF)_2_, Ag_18_(CO_3_)(S-*^t^*Bu)_10_(NO_3_)_6_(DMF)_4_, and Ag_44_(V_10_O_28_)(S-Et)_20_(PhSO_3_)_18_(H_2_O)_2_), the anion template was contained in the center, whereas Au_7_Ag_8_(dppf)_3_(CF_3_COO)_7_ had an icosahedral metal core composed of Au_7_Ag_8_. Such an icosahedral core structure is often seen in metal NCs [27,53]. The 1D CS of [Au_7_Ag_9_(dppf)_3_(CF_3_COO)_7_BF_4_]*_n_* was synthesized in one pot. Probably, excess Ag binds to CF_3_COO^−^ in the ligand layer during synthesis, resulting in the formation of a 1D CS composed of Au_7_Ag_8_(dppf)_3_(CF_3_COO)_7_ NCs. The researchers also revealed that this 1D CS possessed a band gap of 2.18 eV (Figure 10B).

### 2.3. Control of Counterions

**Figure 11:**
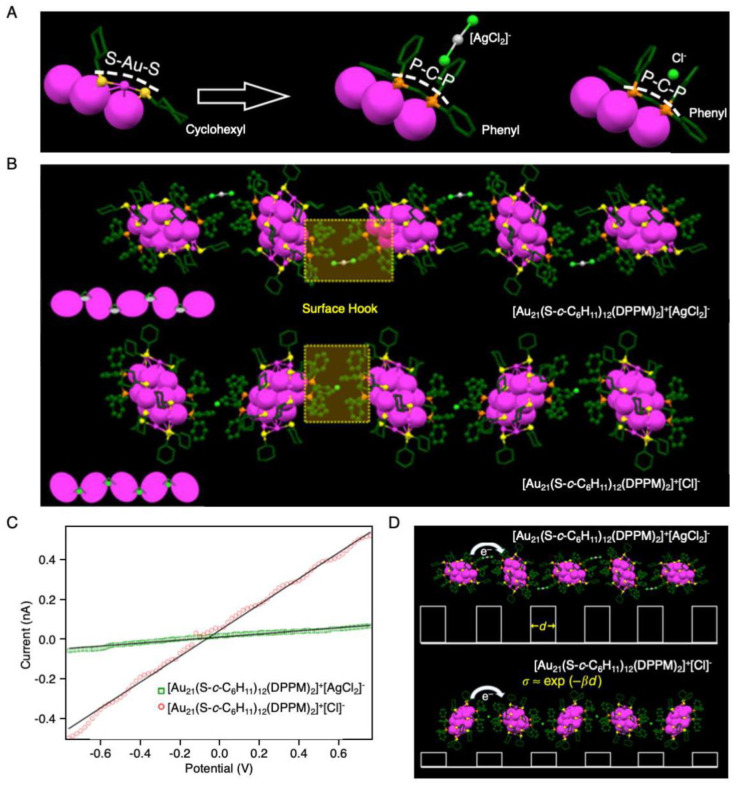
(**A**) Site-specific tailoring of the surface motifs and associated counterions of Au NCs. The two RS-Au-SR (R is cyclohexyl) surface motifs in Au_23_ (precursor NC) were replaced by two DPPM motifs in Au_21_. Each P atom was connected to two phenyl rings. Dashed lines indicate the motifs. (**B**) Packing of [Au_21_(S-*c*-C_6_H_11_)_12_(DPPM)_2_]^+^[AgCl_2_]^−^ and [Au_21_(S-*c*-C_6_H_11_)_12_(DPPM)_2_]^+^[Cl]^−^ in their 1D assemblies. The orientation of Au NCs is modulated by the counterion. Au = magenta, Ag = gray, Cl = light green, S = yellow, P = orange, C = green. All H atoms are omitted for clarity. Yellow areas are the surface hooks connecting neighboring NCs. (**C**) Room-temperature conductivity of single crystals of [Au_21_(S-*c*-C_6_H_11_)_12_(DPPM)_2_]^+^[AgCl_2_]^−^ (green) and [Au_21_(S-*c*-C_6_H_11_)_12_(DPPM)_2_]^+^[Cl]^−^ (red). (**D**) Schematic diagram of electron hopping in Au_21_ NC assemblies. Different configurations of the interacting π–π pairs result in tunneling barriers of different heights (white solid squares), thus changing the electron conductivity (e^−^ represents an electron, σ is the conductivity, *d* is the interparticle distance, and *β* is the tunneling decay constant). Reproduced with permission from Reference [152]. Copyright 2018 Springer-Nature.

For certain metal NCs, the total number of valence electrons satisfies the number for the closed-shell electronic structure when it is a cation, so they are generated as a cation [151]. For example, [Au_21_(S-*c*-C_6_H_11_)_12_(DPPM)]^+^ (S-*c*-C_6_H_11_ = cyclohexanethiolate, Scheme 1(13), and DPPM = bis(diphenylphosphinomethane), Scheme 1(14)) is synthesized as a cation. In 2018, Jin et al. [152] revealed that [Au_21_(S-*c*-C_6_H_11_)_12_(DPPM)]^+^ formed a 1D CS in a crystal by assembling as a pair with the counter anion and that the structure of the 1D CS changed depending on the counterion (Figure 11A,B).

In this study, [Au_21_(S-*c*-C_6_H_11_)_12_(DPPM)_2_]^+^[AgCl_2_]^−^ and [Au_21_(S-*c*-C_6_H_11_)_12_(DPPM)_2_]^+^[Cl]^−^ were precisely synthesized and single crystals were grown. As shown in Figure 11B, a 1D CS was formed by the alternating connection of [Au_21_(S-*c*-C_6_H_11_)_12_(DPPM)_2_]^+^ and [AgCl_2_]^−^ in the [Au_21_(S-*c*-C_6_H_11_)_12_(DPPM)_2_]^+^[AgCl_2_]^−^ crystal. This 1D CS was considered to assemble via π-π, anion-π, and aryl C-H···Cl interactions. The connection pattern of [Au_21_(S-*c*-C_6_H_11_)_12_(DPPM)_2_]^+^ in the 1D CS changed slightly when the counterion was Cl^−^ rather than [AgCl_2_]^−^. It was considered that the connection pattern of [Au_21_(S-*c*-C_6_H_11_)_12_(DPPM)_2_]^+^ changed because the arrangement of phenyl groups in the NC was affected by the counterion (Figure 11A).

The obtained 1D CSs of [Au_21_(S-*c*-C_6_H_11_)_12_(DPPM)_2_]^+^ had different electron transport properties depending on the counter anion. The 1D CS of [Au_21_(S-*c*-C_6_H_11_)_12_(DPPM)_2_]^+^[AgCl_2_]^−^ had a σ of only ~1.44 × 10^−8^ S/m, whereas that of [Au_21_(S-*c*-C_6_H_11_)_12_(DPPM)_2_]^+^[Cl]^−^ was σ ~2.38 × 10^−6^ S/m (Figure 11C). Changing the counter anion from [AgCl_2_]^−^ to [Cl]^−^ shortened the distance between NCs from 16.80 to 16.39 Å and formed an intra-cluster π-stacking structure that allowed electricity to flow easily (Figure 11B). These two reasons explained why σ of [Au_21_(S-*c*-C_6_H_11_)_12_(DPPM)_2_]^+^[Cl]^−^ was two orders of magnitude higher than that of [Au_21_(S-*c*-C_6_H_11_)_12_(DPPM)_2_]^+^[AgCl_2_]^−^ (Figure 11D).

**Figure 12:**
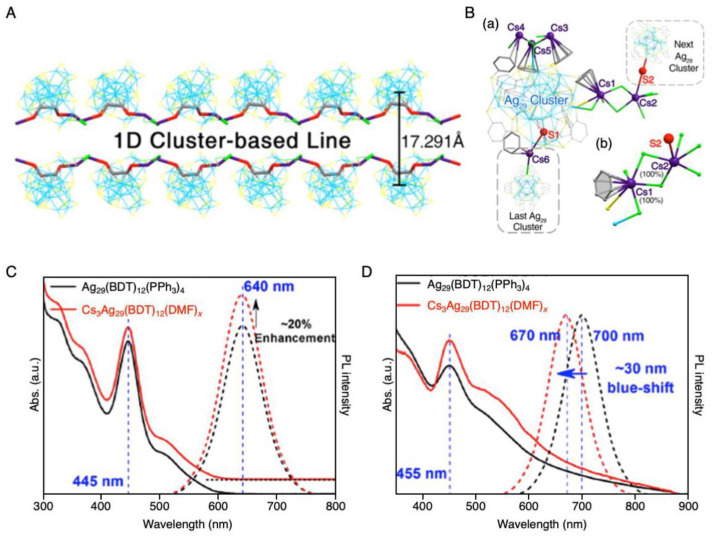
(**A**) 1D linear assembly of [Cs_3_Ag_29_(BDT)_12_(DMF)*_x_*]^0^ in the crystal lattice. Ag = light blue/gray, Cs = dark purple, S = yellow and red, O = green. For clarity, all H, C, and N atoms, some Cs^+^, and DMF molecules are omitted. Each O atom represents a DMF molecule. (**B**) (a) Overall surface structure of [Cs_3_Ag_29_(BDT)_12_(DMF)*_x_*]^0^ and (b) interactions between Ag_29_(BDT)_12_, Cs_1_, Cs_2_, and DMF. (**C**) Comparison of optical absorption and emission spectra of Ag_29_(BDT)_12_(PPh_3_)_4_ (black) and Cs_3_Ag_29_(BDT)_12_(DMF)*_x_* (red) NCs dissolved in DMF. (**D**) Comparison of optical absorption and emission spectra of Ag_29_(BDT)_12_(PPh_3_)_4_ (black) and Cs_3_Ag_29_(BDT)_12_(DMF)*_x_* (red) NCs in crystalline films. Reproduced with permission from Reference [154]. Copyright 2019 American Chemical Society.

There are not only metal NCs synthesized as cations but also metal NCs synthesized as anions. Because the total number of valence electrons of [Ag_29_(BDT)_12_(PPh_3_)_4_]^3−^ (BDT = 1,3-benzenedithiolate, Scheme 1(15); PPh_3_ = triphenylphosphine, Scheme 1(16)) satisfies the number for a closed-shell electronic structure as the anion, it is generated as the anion [153]. In 2019, Zhu et al. [154] reported that mixing this NC with Cs acetate in DMF induced Cs^+^ attachment to the NC and PPh_3_ desorption from the NC, resulting in the formation of [Cs_3_Ag_29_(BDT)_12_(DMF)*_x_*]^0^ (*x* = 5 or 6) and that the obtained [Cs_3_Ag_29_(BDT)_12_(DMF)*_x_*]^0^ formed a 1D CS in its crystal. Figure 12A shows the resulting 1D CS. In the crystal, [Cs_3_Ag_29_(BDT)_12_(DMF)*_x_*]^0^ was connected by a series of bonds consisting of −Cs^+^−DMF−Cs^+^−S−Ag−Ag−S−. This 1D CS was considered to be formed because of the electrostatic attraction between [Ag_29_(BDT)_12_(DMF)*_x_*]^3−^ and Cs^+^, Cs−S bond formation, and Cs···π interactions (Figure 12B).

Both [Ag_29_(BDT)_12_(PPh_3_)_4_]^3−^ and [Cs_3_Ag_29_(BDT)_12_(DMF)*_x_*]^0^ solutions showed similar absorption and photoluminescence (PL) spectra (Figure 12C). This indicates that Cs^+^ attachment and PPh_3_ desorption did not markedly change the electronic structure of the Ag_29_(BDT)_12_ NCs. In contrast, the absorption and PL spectra of [Ag_29_(BDT)_12_(PPh_3_)_4_]^3-^ and [Cs_3_Ag_29_(BDT)_12_(DMF)*_x_*]^0^ in the crystalline state were quite different (Figure 12D). The 1D CS of [Cs_3_Ag_29_(BDT)_12_(DMF)*_x_*]^0^ was considered to show different optical behavior from that of the individual [Ag_29_(BDT)_12_(PPh_3_)_4_]^3−^ because of the electronic interactions between adjacent NCs in the 1D CS of [Cs_3_Ag_29_(BDT)_12_(DMF)*_x_*]^0^.

### 2.4. Introduction of Linker Molecules

In the examples described in Section 2.3, 1D CSs were formed by the counterion acting as a linker. When an organic molecule is used as the linker, the distance between NCs in a 1D CS can be freely controlled because the design of the structure of organic molecules is well understood. In fact, the geometry of an MOF is controlled by the design of the linker organic molecule [155]. In recent years, several 1D CSs of metal NCs with organic molecules as linkers have also been reported.

**Figure 13:**
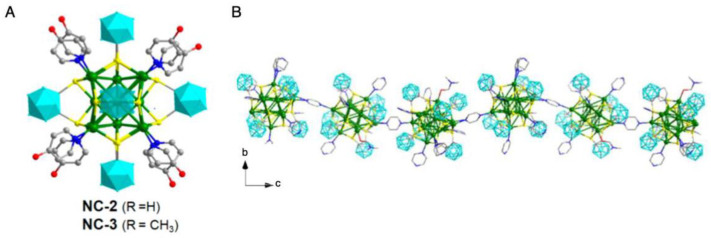
(**A**) Structure of Ag_14_(DT-*o*-C)_6_(pyridine/*p*-methylpyridine)_8_. (**B**) 1D helix of Ag_14_(DT-*o*-C)_6_ NC. Ag = green and pink, S = yellow, C = gray, N = blue, carborane = turquoise. Reproduced with permission from Reference [156]. Copyright 2018 American Chemical Society.

For example, in 2018, Zang et al. [156] reported a 1D CS in which Ag_14_(DT-*o*-C)_6_ NCs (DT-*o*-C = 1,2-dithiolate-o-carborane, Scheme 1(17)) were linked by pyrazine (Scheme 2(1)). In this study, first, [Ag_14_(DT-*o*-C)_6_(pyridine/*p*-methylpyridine)_8_] (Scheme 2(2),(3)) were identified as Ag NCs with high thermal stability that maintained their framework structure even at 150 °C or higher in air (Figure 13A). Then, they attempted to synthesize Ag NCs in which the pyridine or *p*-methylpyridine ligands of these Ag NCs were replaced by pyrazine. As a result, they obtained a 1D CS in which Ag_14_(DT-*o*-C)_6_ NCs were linked by pyrazine (Figure 13B). In the obtained structure, pyrazine was coordinated to each Ag_14_(DT-*o*-C)_6_ NC at a diagonal position, which caused the 1D CS to rotate in the clockwise direction with respect to the (001) axis. In this study, the researchers also succeeded in forming 2D and 3D CSs composed of Ag_14_(DT-*o*-C)_6_ NCs by changing the structure of the bipyridine ligand, as described later in Section 3.2 and Section 4.3, respectively.

**Figure 14:**
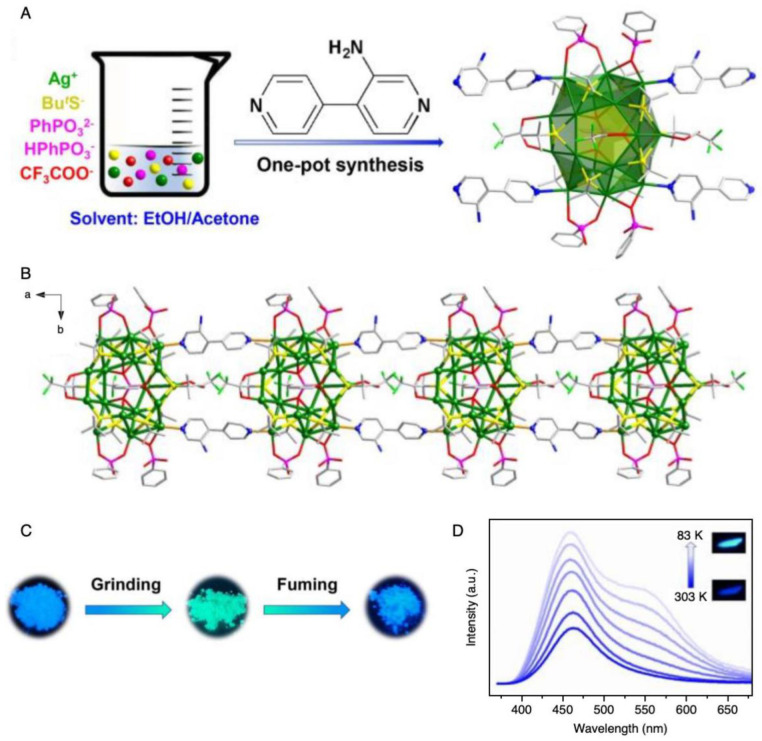
(**A**) Schematic representation of the one-pot synthesis of [Ag_18_(PhPO_3_)(S-*^t^*Bu)_10_(CF_3_COO)_2_(PhPO_3_H)_4_(bpy-NH_2_)_2_]·(PhPO_3_H_2_). (**B**) 1D structure of [Ag_18_(PhPO_3_)(S-*^t^*Bu)_10_(CF_3_COO)_2_(PhPO_3_H)_4_(bpy-NH_2_)_2_]·(PhPO_3_H_2_). Ag = green, S = yellow, C = gray, N = blue, O = red, F = light green, P = purple. H atoms are omitted for clarity. (**C**) Luminescent images of the as-synthesized, ground, and fumed [Ag_18_(PhPO_3_)(S-*^t^*Bu)_10_(CF_3_COO)_2_(PhPO_3_H)_4_(bpy-NH_2_)_2_] under ultraviolet light irradiation. (**D**) Temperature-dependent luminescence spectra of [Ag_18_(PhPO_3_)(S-*^t^*Bu)_10_(CF_3_COO)_2_(PhPO_3_H)_4_(bpy-NH_2_)_2_] from 30 to −190 °C in the solid state. The inset photographs show the emission of [Ag_18_(PhPO_3_)(S-*^t^*Bu)_10_(CF_3_COO)_2_(PhPO_3_H)_4_(bpy-NH_2_)_2_] in the solid state under ultraviolet light irradiation at room temperature and liquid nitrogen temperature. Reproduced with permission from Reference [157]. Copyright 2019 Wiley-VCH.

Because N readily coordinates to Ag, bipyridine is often used to connect NCs containing Ag. In 2019, Zang and colleagues formed a 1D CS composed of Ag_18_(PhPO_3_)(S-*^t^*Bu)_10_(CF_3_COO)_2_(PhPO_3_H)_4_ (PhPO_3_^2−^ = phenylphosphinic diion; PhPO_3_H^−^ = phenylphosphinic acid ion) NC nodes with bipyridine(3-amino-4,4′-bipyridine (bpy-NH_2_, Scheme 2(4)) linkers [157]. In this experiment, NC synthesis, ligation, and crystallization were performed simultaneously in one pot (Figure 14A). The node Ag_18_(PhPO_3_)(S-*^t^*Bu)_10_ NCs contained PhPO_3_^2−^ as an anion template in their center. In the crystal, adjacent Ag_18_(PhPO_3_)(S-*^t^*Bu)_10_ NCs were linked by two bpy-NH_2_ to form a 1D CS (Figure 14B).

The obtained 1D CS was stable up to 110 °C in a nitrogen (N_2_) atmosphere. When mechanical stimulation was applied to the 1D CS, its PL wavelength changed. When the 1D CS sample subjected to mechanical stimulation was recrystallized, its PL wavelength returned to the original value (Figure 14C). Thus, the PL of the 1D CS composed of Ag_18_(PhPO_3_)(S-*^t^*Bu)_10_ NCs exhibited reversible mechanochromism. Because this 1D CS emitted light at two wavelengths and its PL intensity ratio changed with temperature (thermochromism; Figure 14D), the authors suggested that this 1D CS could be applied as a thermometer.

**Figure 15:**
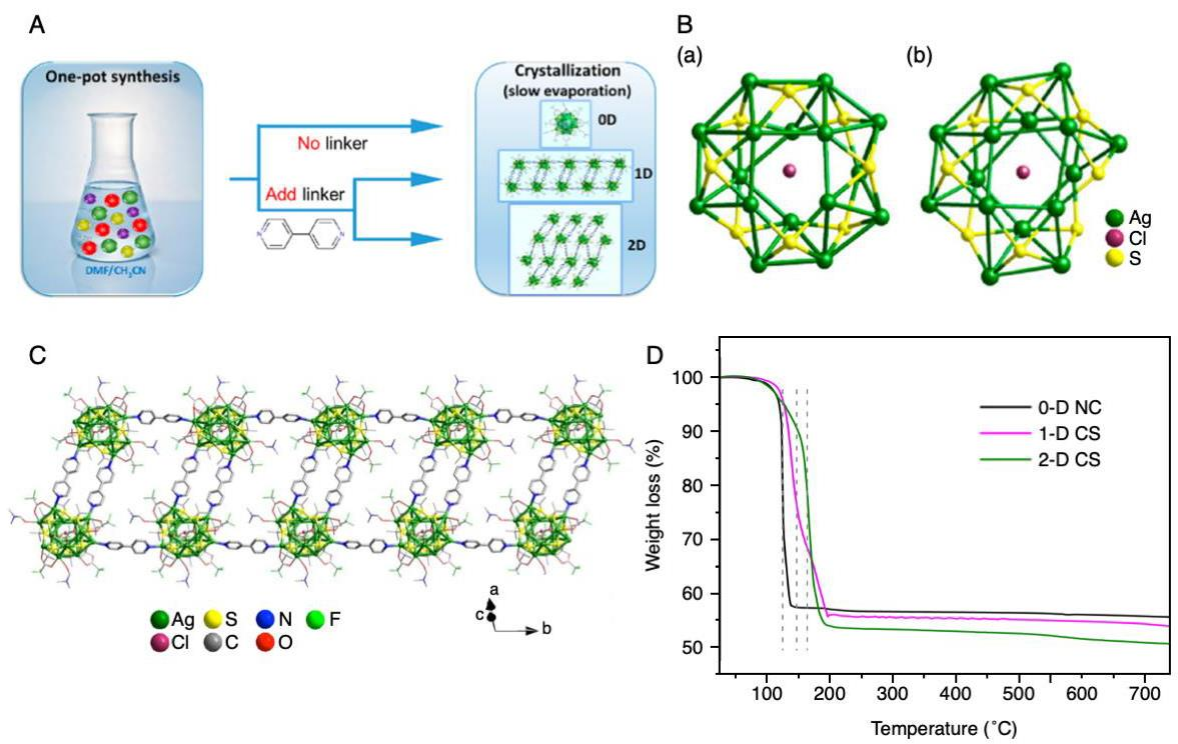
(**A**) Synthesis of Ag NCs and NC-based frameworks. (**B**) Top views of the core structures of (a) Ag_16_Cl(S-*^t^*Bu)_8_(CF_3_COO)_7_(DMF)_4_(H_2_O) and (b) Ag_15_Cl(S-*^t^*Bu)_8_(CF_3_COO)_5.67_(NO_3_)_0.33_(DMF)_2_. (**C**) Crystal structure of the corresponding 1D CS. Free (co-crystallized) DMF molecules are not shown. The green semitransparent spheres in the Ag clusters are shown as a visual guide. H atoms were omitted for clarity. (**D**) Thermogravimetric analysis curves of NCs, 1D CS, and 2D CS (see Section 3.2). Reproduced with permission from Reference [158]. Copyright 2019 American Chemical Society.

In 2019, Bakr et al. [158] also reported the connection of Ag NCs by bipyridine. In this study, a 1D CS was synthesized in one pot (Figure 15A). Ag_15_Cl(S-*^t^*Bu)_8_(CF_3_COO)_5.67_(NO_3_)_0.33_(DMF)_2_ was used as the node, and 4,4’-bipyridine (bpy, Scheme 2(5)) was used as the linker. In Ag_15_Cl(S-*^t^*Bu)_8_(CF_3_COO)_5.67_(NO_3_)_0.33_(DMF)_2_, Cl^−^acted as an anion template. The core of the Ag_15_Cl NC had a geometry in which one Ag was lost from the Ag_16_Cl core of the Ag_16_Cl(S-*^t^*Bu)_8_(CF_3_COO)_7_(DMF)_4_(H_2_O) NC, which did not form a 1D CS, namely individual Ag_16_Cl(S-*^t^*Bu)_8_(CF_3_COO)_7_(DMF)_4_(H_2_O) NCs (Figure 15B). The 1D CS with a ladder structure was formed by combining these Ag_15_Cl NCs with three adjacent Ag_15_Cl NCs via four bpy molecules (Figure 15C). It was found that the 1D CS displayed slightly higher thermal stability than that of the Ag_16_Cl NCs (Figure 15D).

**Figure 16:**
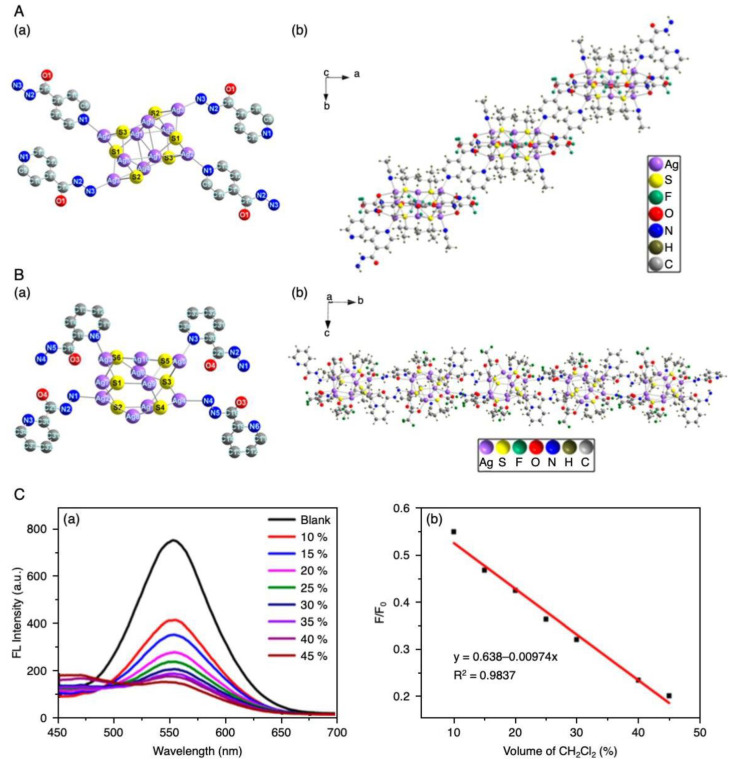
(a) Structural units and (b) spatial stacking diagrams of (**A**) Ag_10_(CF_3_COO)_4_(S-*^t^*Bu)_6_(CH_3_CN)_2_(*p*-iah)_2_ and (**B**) Ag_10_(CF_3_COO)_4_(S-*^t^*Bu)_6_(CH_3_CN)(*o*-iah)_2_. (**C**) (a) PL spectra of Ag_10_(CF_3_COO)_4_(S-*^t^*Bu)_6_(CH_3_CN)_2_(*o*-iah)_4_ in CCl_4_ with various volume fractions of CH_2_Cl_2_. (b) Linear plot of fluorescence intensity against the volume fraction of CH_2_Cl_2_ in CCl_4_. Reproduced with permission from Reference [159]. Copyright 2019 American Chemical Society.

In the above three studies, the bipyridines had a rigid framework structure. In 2019, Cao et al. formed 1D CSs of Ag NCs by using pyridine derivatives (*p*-iah = 4-pyridine carboxylic hydrazide, Scheme 2(6); *o*-iah = 2-carboxylic hydrazide, Scheme 2(7)) that contained N in both the rigid pyridine framework and flexible substituents [159]. In this study, the 1D CSs were obtained by reacting Ag_12_(S-*^t^*Bu)_6_(CF_3_COO)_6_(CH_3_CN)_6_ (CH_3_CN = acetonitrile) with the above-mentioned pyridine derivatives. The SC-XRD analysis of the products revealed that Ag_10_(CF_3_COO)_4_(S-*^t^*Bu)_6_(CH_3_CN)_2_ and Ag_10_(CF_3_COO)_4_(S-*^t^*Bu)_6_(CH_3_CN) were the nodes in the 1D CSs with *p*-iah and *o*-iah, respectively (Figure 16A(a),B(a)). These 1D CSs containing *p*-iah and *o*-iah had cross-helical and parallel chain structures, respectively (Figure 16A(b),B(b)). The latter structure also contained hydrogen bonds (N−H···O) between the parallel linker molecules. It is interesting that different 1D CSs formed depending on the position of N in the linker molecule (Scheme 2(6),(7)).

Both 1D CSs showed PL and that with *o*-iah as the linker exhibited weak green PL in highly polar solvents and strong yellow PL in solvents with low polarity. Based on these characteristics, the authors suggested that the 1D CS with *o*-iah could be used to measure the concentration of dichloromethane (CH_2_Cl_2_, Figure 16C) or trichloromethane (CHCl_3_) in tetrachloromethane (CCl_4_).

**Figure 17:**
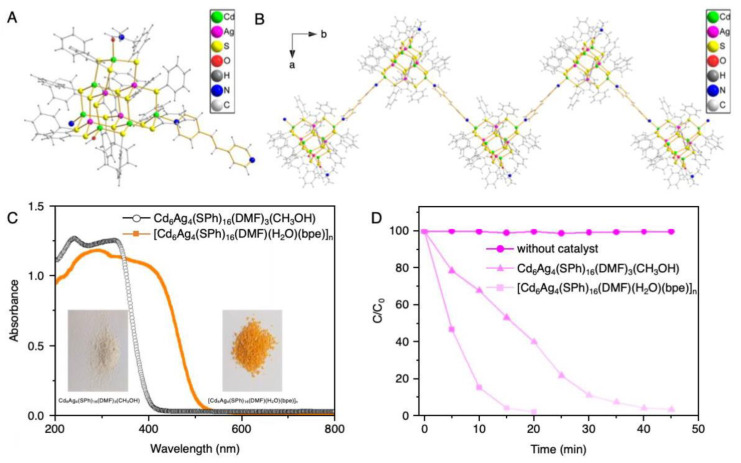
(**A**) Structures of Cd_6_Ag_4_(S-Ph)_16_(DMF)(H_2_O) and (**B**) 1D CS of Cd_6_Ag_4_(S-Ph)_16_(DMF)(H_2_O)(bpe). (**C**) Solid-state ultraviolet-visible diffuse reflectance spectra of the discrete Cd_6_Ag_4_(SPh)_16_(DMF)_3_(CH_3_OH) (open circles) and 1D CS of Cd_6_Ag_4_(S-Ph)_16_(DMF)(H_2_O)(bpe) (filled squares). (**D**) Comparison of the photocatalytic-degradation efficiencies of the discrete Cd_6_Ag_4_(S-Ph)_16_(DMF)_3_(CH_3_OH) (triangles), 1D CS of Cd_6_Ag_4_(S-Ph)_16_(DMF)(H_2_O)(bpe) (squares), and without a catalyst (circles). Reproduced with permission from Reference [160]. Copyright 2020 American Chemical Society.

Bipyridines can also be used as linkers to form 1D CSs of Ag chalcogenide NCs. Very recently, Xu and co-workers reported the formation of a zigzag-type of 1D CS with Cd_6_Ag_4_(S-Ph)_16_(DMF)(H_2_O) (S-Ph = benzenethiolate, Scheme 1(18) and Figure 17A) as a node and *trans*-1,2-bis(4-pyridyl)ethylene (bpe, Scheme 2(8)) as a linker (Figure 17B) [160]. This 1D CS was obtained by the reaction of Cd_6_Ag_4_(S-Ph)_16_(DMF)_3_(CH_3_OH) (CH_3_OH = methanol) with bpe. In the obtained 1D CS, N of bpe coordinated to Cd not Ag (Figure 17A). Such a coordination pattern has also been observed in 2D and 3D CSs composed of Cd_6_Ag_4_(S-Ph)_16_ and bpe previously reported by Zhang et al. [161,162].

They compared the electronic structures of the resulting 1D CS and individual Cd_6_Ag_4_(S-Ph)_16_(DMF)_3_(CH_3_OH) NCs. The results revealed that the band gap of the NCs was narrowed by the formation of the 1D CS (Figure 17C). In the 1D CS, the optical absorption onset was redshifted to the visible region. They used the 1D CS as a visible light (>420 nm)-responsive photocatalyst to decompose the organic dye Rhodamine B in water. The 1D CS exhibited higher photocatalytic activity toward Rhodamine B degradation than that of the Cd_6_Ag_4_(S-Ph)_16_ NCs (Figure 17D) and high stability during the photocatalytic reaction.

## 3. Two-Dimensional Structures

To date, Ag NCs have been used as the building blocks in almost all 2D CSs. Like Au NCs, Ag NCs have unique electronic and optical properties [68,163,164,165,166] and are expected to be applied in various fields. However, Ag NCs are less stable than Au NCs against external stimuli, such as light and solvents. Therefore, studies have been actively conducted to improve the stability of Ag NCs by assembly of CSs and thereby improve their physical properties. In Section 3.1 and Section 3.2 we focus on the connection of NCs by Ag−O bond formation (Figure 2B) and introducing linker molecules (Figure 2D), respectively. Table 2 summarizes the connection methods, NCs, linkers, reported years, and reference numbers of the relevant literature. Several of the ligands used in these studies are shown in Scheme 1. The organic molecules used as linkers are depicted in Scheme 2.

### 3.1. Connection via Ag−O Bonds

**Figure 18:**
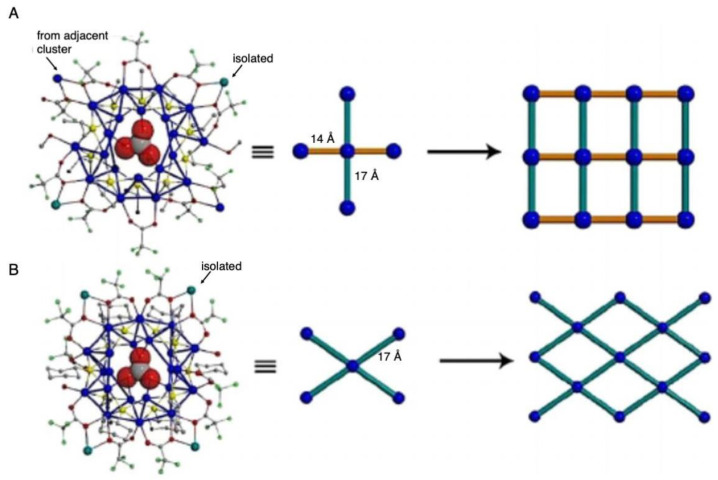
(**A**) The molecular building block in Ag_20_(CO_3_)(S-*i*Pr)_10_(CF_3_COO)_9_(CF_3_COOH)(CH_3_OH)_2_ with its four linking sites, and condensation of blocks into a 4,4-net. (**B**) Similar condensation of molecular building blocks of Ag_20_(CO_3_)(S-*c*-C_6_H_11_)_10_(CF_3_COO)_10_(CF_3_COOH)_2_(H_2_O)_2_. Note the difference between the ‘arms’ with lengths of 14 Å (orange) and 17 Å (cyan). Ag = blue and cyan, S = yellow, O = red, C = gray, F = green. Reproduced with permission from Reference [148]. Copyright 2017 Wiley-VCH.

In 2017, Mak et al. [148] reported the formation of 2D CSs with Ag_20_(CO_3_)(S-*i*Pr)_10_(CF_3_COO)_9_(CF_3_COOH)(CH_3_OH)_2_ (S-*i*Pr = isopropylthiolate, Scheme 1(5)) or Ag_20_(CO_3_)(S-*c*-C_6_H_11_)_10_(CF_3_COO)_10_(CF_3_COOH)_2_(H_2_O)_2_ as building blocks in their paper on the formation of 1D CSs. These NCs contained CO_3_^2−^ as an anion template at the center of their cores (Figure 18A,B). The structures of the SR in these two types of Ag_20_(CO_3_)(SR)_10_ NCs were different (S-*i*Pr vs. S-*c*-C_6_H_11_), which influenced the formation angle and bond distance between adjacent NCs in the 2D CSs. The O^−^ of CF_3_COO^−^ and Ag of an adjacent NC were directly linked via an Ag−O bond with a length of 14 Å. In addition, isolated Ag was trapped between CF_3_COO^−^ of adjacent NCs, so the adjacent NCs were connected via an O−Ag−O bond with a length of 17 Å. The 2D CS consisting of Ag_20_(CO_3_)(S-*i*Pr)_10_(CF_3_COO)_9_(CF_3_COOH)(CH_3_OH)_2_ showed dual emission at room temperature. Because both zero-dimensional [Ag_20_(S-*^t^*Bu)_10_(CF_3_COO)_2_]Cl·(CF_3_COO)_7_·5CH_3_OH NCs and the 1D CS of Ag_20_(CO_3_)(S-*^t^*Bu)_10_(CH_3_COO)_8_(DMF)_2_ emit only single emission peaks, it was speculated that the formation of the 2D CS was related to the observed dual emission.

**Figure 19:**
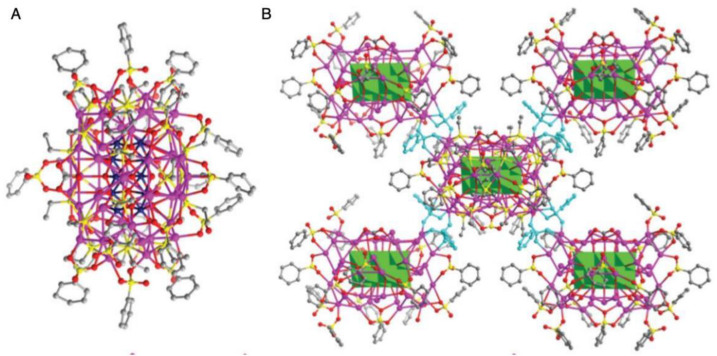
(**A**) Structure and (**B**) 2D extended layer structure of Ag_46_(V_10_O_28_)(S-Et)_23_(PhSO_3_)_15_(CO_3_) with all bridging PhSO_3_^−^ ligands highlighted in cyan and V_10_O_28_^6−^ shown as green polyhedra. Ag = purple, V = dark blue, S = yellow, C = gray, O = red. All H atoms are omitted. Reproduced with permission from Reference [149]. Copyright 2019 Royal Society of Chemistry.

In 2019, Sun and co-workers also reported the formation of a 2D CS, using Ag NCs [149]. In this study, a 2D CS consisting of Ag_46_(V_10_O_28_)(S-Et)_23_(PhSO_3_)_15_(CO_3_) was formed (Figure 19), using a different solvent from the case of Ag_44_(V_10_O_28_)(S-Et)_20_(PhSO_3_)_18_(H_2_O)_2_, which formed a 1D CS (Figure 9). These Ag NCs have the same total number of ligands (S-Et and PhSO_3_^−^) of 38 but different ratios of the ligand types. They considered that the ligand ratio affected the number of Ag atoms in the core and also the connection mode between adjacent NCs.

**Figure 20:**
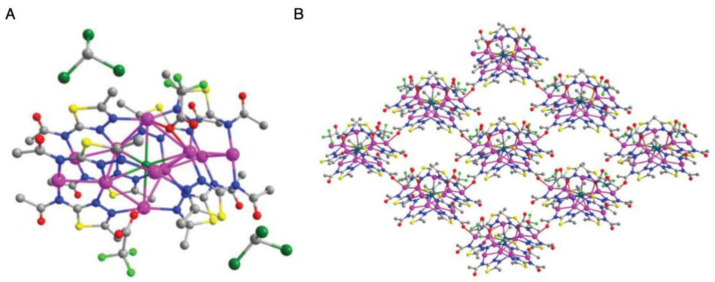
(**A**) Structure of Ag_11_Cl(N-L)_8_(CF_3_COO)_2_·2CHCl_3_ and (**B**) simplified 2D network featuring a four-connected topology. Ag = purple, S = yellow, O = red, C = gray, Cl = dark green, N = blue, F = neon green. Reproduced with permission from Reference [166]. Copyright 2019 Royal Society of Chemistry.

Xu et al. [166], also in 2019, reported 2D CSs of Ag_11_Cl(N-L)_8_(CF_3_COO)_2_·2CHCl_3_ (Figure 20), Ag_11_Cl(N-L)_8_(NO_3_)_2_·2CHCl_3_, and Ag_11_Cl(N-L)_8_(CF_3_SO_3_)_2_·2CHCl_3_ (N-L = 2-acetamido-5-methyl-1,3,4- thiadiazole, Scheme 1(19)), in which adjacent NCs are linked by Ag−O bonds. SR, alkyne, or phosphine ligands are generally used in metal NCs. In this study, their aim was to synthesize Ag NCs by using an N-donor ligand, which is not appropriate based on the hard/soft acid/base theory [167], and form corresponding CSs. The three kinds of 2D CSs obtained had similar frameworks regardless of the coordination ions (CF_3_COO^−^, NO_3_^−^, or CF_3_SO_3_^−^), which means that the framework structure shown in Figure 20B is very rigid. It was found that Ag_11_Cl(N-L)_8_(CF_3_COO)_2_·2CHCl_3_ showed dual-emission behavior and that its PL peaks had different optimal excitation wavelengths.

### 3.2. Introduction of Linker Molecules

**Figure 21:**
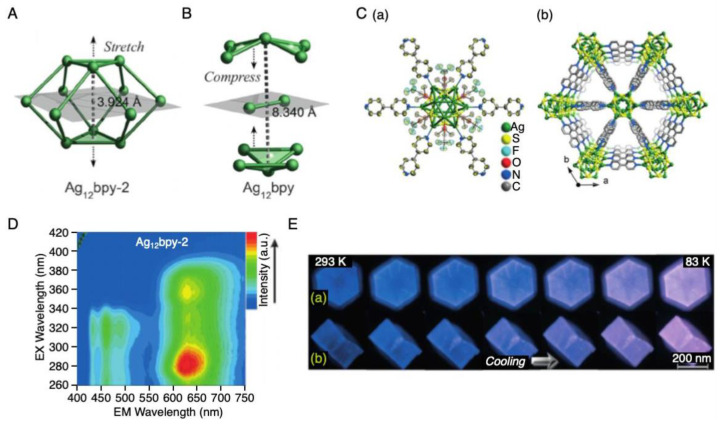
Comparison of the Ag_12_ core structures in (**A**) Ag_12_(S-*^t^*Bu)_6_(CF_3_COO)_6_(bpy)_3_ (Ag_12_bpy-2) and (**B**) Ag_12_(S-*^t^*Bu)_8_(CF_3_COO)_4_(bpy)_4_ (Ag_12_bpy). (**C**) (a) Perspective view of an Ag_12_S_6_ node with six pendant bpy linkers (ORTEP drawing at the 50% probability level). (b) Stacking of the 2D network structure of Ag_12_(S-*^t^*Bu)_6_(CF_3_COO)_6_(bpy)_3_ viewed along the crystallographic *c*-axis. Ag = green, S = yellow, C = gray, N = blue; CF_3_COO^−^, *^t^*Bu, and H atoms are omitted for clarity. (**D**) 3D-excitation emission matrix of Ag_12_(S-*^t^*Bu)_6_(CF_3_COO)_6_(bpy)_3_ measured at −190 °C. (**E**) Thermochromic images of the (a) exterior {001} surfaces and (b) exposed interior {010}/{100} planes of Ag_12_(S-*^t^*Bu)_6_(CF_3_COO)_6_(bpy)_3_/NH_2_⋅(bpy:bpy-NH_2_ = 20:1) solvated single crystals under ultraviolet light irradiation. Reproduced with permission from Reference [169]. Copyright 2018 Wiley-VCH.

As described below, in Section 4.3, in 2017, Zang et al. [168] reported the formation of a 3D CS in which Ag_12_(S-*^t^*Bu)_8_(CF_3_COO)_4_ NCs were linked by bpy. This was a pioneering study on the formation of an MOF, using Ag NCs as nodes, and has greatly influenced subsequent studies. In 2018, they synthesized a 2D CS consisting of Ag_12_ NCs and bpy [169]. The core structure of the node was changed (isomerized) by dissolving an Ag_12_(S-*^t^*Bu)_6_(CF_3_COO)_6_ NC MOF crystal in a mixed solvent consisting of *N,N*-dimethylethanamide (DMAC) and toluene, which changed the geometrical structure of the entire CS from 3D to 2D (Figure 21A,B). This result indicates that the solvent selection is important in the design of the structure of metal NCs and their CSs. In the 2D CS consisting of newly formed Ag_12_(S-*^t^*Bu)_6_(CF_3_COO)_6_ NCs, each Ag_12_(S-*^t^*Bu)_6_(CF_3_COO)_6_ NC was linked to six adjacent Ag_12_(S-*^t^*Bu)_6_(CF_3_COO)_6_ NCs via linkers to produce a highly symmetric 2D CS (Figure 21C). The layers were separated by 7.23 Å, with weak interactions between them. It was also revealed that the reversible structural transformation between 3D and 2D CSs could be induced by appropriate solvent selection (Figure 21A,B).

Structural deformation of the CSs also induced changes in their electronic structure and PL properties. For example, the 3D CS only showed PL at a single wavelength, regardless of temperature and excitation wavelength, whereas the 2D CS exhibited PL of two colors (blue and red), depending on the excitation wavelength (Figure 21D). To enhance the blue emission of the 2D CS, the researchers introduced bpy-NH_2_, which itself emits blue light, as a linker, to fabricate a 2D CS containing two types of linkers, bpy and bpy-NH_2_. The intensity ratio of the red and blue PL signals depended on the mixing ratio of linker molecules. At the optimum linker mixing ratio, the PL intensity ratio of the red and blue peaks depended on temperature. Therefore, this 2D CS containing two types of linkers could be used as a temperature sensor (Figure 21E).

**Figure 22:**
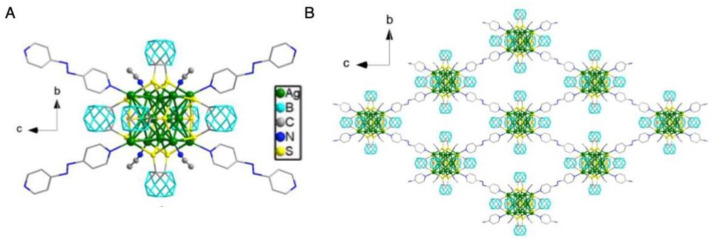
(**A**) Structural unit and (**B**) 2D CS of Ag_14_(DT-*o*-C)_6_(CH_3_CN)_4_(dipyridin-4-yl-diazene)_2_. Reproduced with permission from Reference [156]. Copyright 2018 American Chemical Society.

In 2018, the same group also reported the formation of a 2D CS consisting of Ag_14_(DT-*o*-C)_6_ NCs [156]. The 2D CS with Ag_14_(DT-*o*-C)_6_, as a structural unit (Figure 22A), was fabricated by changing the linker structure from that used to form the 1D CS (Section 2.4 and Figure 13) to dipyridin-4-yl-diazene (Scheme 2(9)). The obtained 2D CS possessed a rhombic network structure with Ag_14_(DT-*o*-C)_6_(CH_3_CN)_4_ as nodes (Figure 22B).

**Figure 23:**
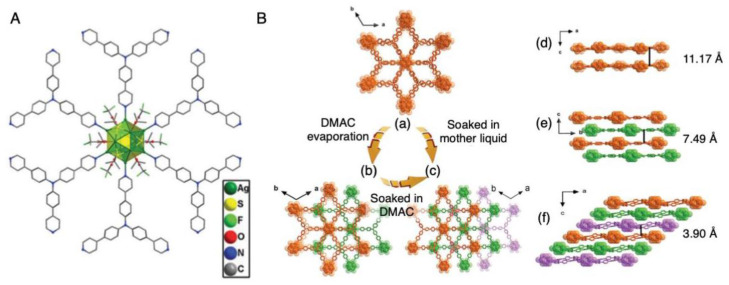
(**A**) Perspective view of an Ag_12_S_6_ subunit with six pendant TPPA linkers in each layer. (**B**) Stacking of the 2D network structure of Ag_12_(S-*^t^*Bu)_6_(CF_3_COO)_6_ by TPPA. AA stacking viewed along the (a) *c*-axis and (d) *b*-axis, AB stacking viewed along the (b) *c*-axis and (e) *a*-axis, and ABC stacking viewed along the (c) *c*-axis and (f) *a*-axis. Reproduced with permission from Reference [170]. Copyright 2018 Royal Society of Chemistry.

In 2018, Zang et al. [170] reported the formation of a 2D CS consisting of Ag_12_(S-*^t^*Bu)_6_(CF_3_COO)_6_ and tris(4-pyridylphenyl)-amine (TPPA; Scheme 2(10) and Figure 23A). This structure is interesting because the distance between the 2D layers can be changed. In this 2D CS, DMAC used as a solvent existed between layers immediately after the synthesis and the 2D layers overlapped, as shown in Figure 23B(a),(d). When the DMAC was partially removed from this structure, the overlap of the 2D layers changed, as illustrated in Figure 23B(b),(e). Furthermore, when this structure was immersed again in the mother liquor, the structure changed to that depicted in Figure 23B(c),(f). It was also found that the size of the crystal and its emission characteristics changed in accordance with the overlap manner in the 2D CS.

**Figure 24:**
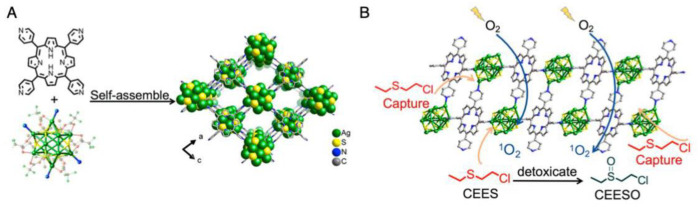
(**A**) Synthesis of Ag_12_(S-*^t^*Bu)_6_(CF_3_COO)_3_(TPyP). (**B**) Schematic illustration of the capture and photodetoxification of CEES by Ag_12_(S-*^t^*Bu)_6_(CF_3_COO)_3_(TPyP). Reproduced with permission from Reference [171]. Copyright 2019 American Chemical Society.

Recently, this group also formed a 2D CS with Ag_12_(S*^t^*Bu)_6_(CF_3_COO)_3_ NCs as nodes by using 5,10,15,20-tetra(4-pyridyl)porphyrin (TPyP, Scheme 2(11)) as a linker (Figure 24A) [171]. TPyP has a photosensitizing effect. Therefore, the ability of the 2D CS to degrade the toxic substance 2-chloroethyl ethyl sulfide (CEES), also called mustard gas, was studied (Figure 24B). The obtained 2D CS showed higher photocatalytic activity than that of a reported MOF. This high photocatalytic activity was ascribed to the synergistic effect of Ag NCs and TPyP, promoting the production of singlet oxygen, which induced the degradation of CEES (Figure 24B). The 2D CS maintained its crystallinity after the photocatalytic reaction and was able to be used repeatedly. The authors pointed out that photocatalytic activity could be further increased by selecting appropriate Ag NCs and organic molecular linkers.

**Figure 25:**
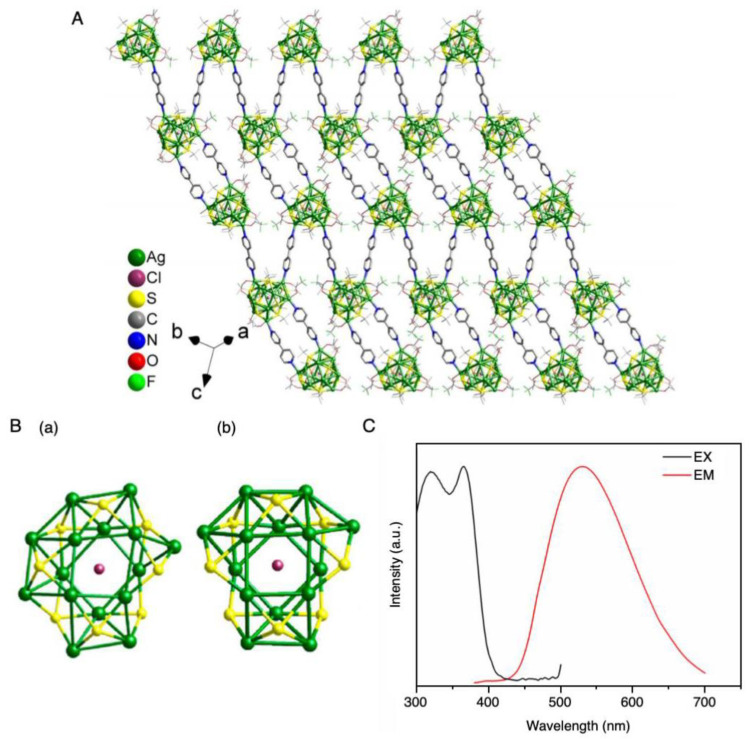
(**A**) Structure of Ag_14_Cl(S-*^t^*Bu)_8_(CF_3_COO)_5_(DMF)(bpy)_2_. Free (co-crystallized) DMF molecules are not shown. (**B**) Top views of the core structures of (a) Ag_15_Cl(S-*^t^*Bu)_8_(CF_3_COO)_5.67_(NO_3_)_0.33_(DMF)_2_ and (b) Ag_14_Cl(S-*^t^*Bu)_8_(CF_3_COO)_5_(DMF). The green semitransparent spheres in the Ag NCs are shown as a visual guide. H atoms have been omitted for clarity. (**C**) Steady-state PL and excitation spectra of 2D CS crystals measured at room temperature (~25 °C). Emission spectra were measured under 365 nm excitation. Reproduced with permission from Reference [158]. Copyright 2019 American Chemical Society.

**Figure 26:**
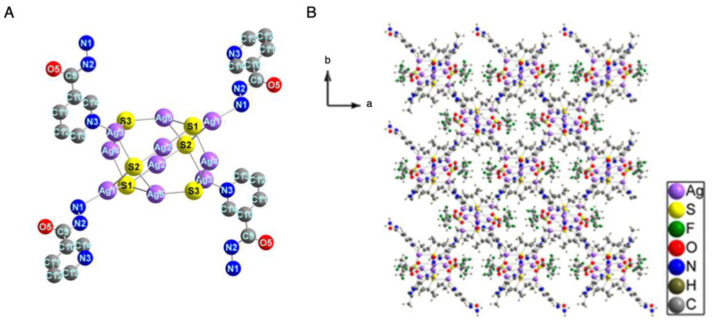
(**A**) Structural unit and (**B**) spatial stacking diagram of Ag_10_(CF_3_COO)_4_(S-*^t^*Bu)_6_(CH_3_CN)_4_(*m*-iah)_4_. Reproduced with permission from Reference [159]. Copyright 2019 American Chemical Society.

Two other types of 2D CSs were also reported in 2019. In their paper on 1D CS formation (Figure 15 and Section 2.4), Bakr et al. [158] also reported that a 2D CS with Ag_14_Cl(S-*^t^*Bu)_8_(CF_3_COO)_5_(DMF) as nodes was formed by changing the concentration of bpy during synthesis (Figure 25A). This Ag_14_Cl core contained Cl^−^ at its center as an anion template. Compared with the Ag_15_Cl(S-*^t^*Bu)_8_(CF_3_COO)_5.67_(NO_3_)_0.33_(DMF)_2_ node of the 1D CS, the node of the 2D CS (Ag_14_Cl(S-*^t^*Bu)_8_(CF_3_COO)_5_(DMF)) had one less Ag atom. However, the frameworks of these NCs were similar to each other (Figure 25B). The 2D CS showed higher thermal stability than those of individual Ag NCs and the 1D CS (Figure 15D). Unlike individual Ag NCs and the 1D CS, the 2D CS emitted green light with a strong intensity, even at room temperature (Figure 25C). Based on the results of a DFT calculation, it was interpreted that the enhancement of PL intensity was caused by a linker-to-cluster charge transfer excitation. In addition, Gao et al. [159] recently formed a 2D CS (Figure 26) with Ag_10_(CF_3_COO)_4_(S-*^t^*Bu)_6_(CH_3_CN)_4_ nodes, using 3-pyridine carboxylic hydrazide (*m*-iah, Scheme 2(12)) as a linker, which is the *meta* equivalent of *p*-iah and *o*-iah used to form 1D CSs (Figure 16).

**Figure 27:**
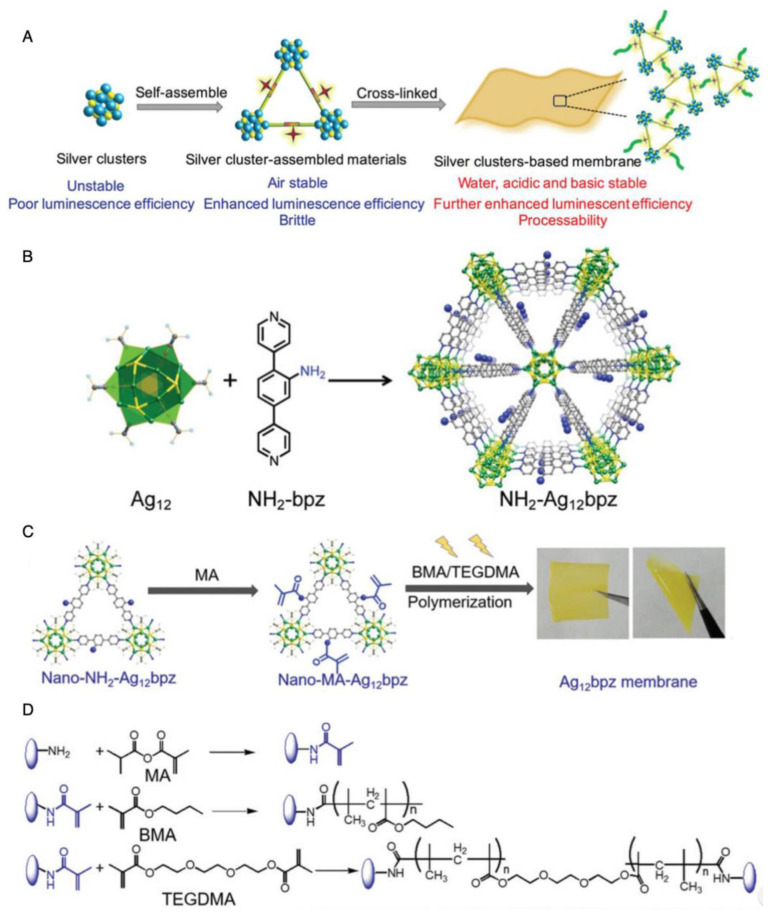
(**A**) Schematic illustration of the fabrication process of an Ag NC-based membrane. (**B**) Structure views of Ag_12_(S-*^t^*Bu)_6_(CF_3_COO)_6_(bpz-NH_2_)_3_. (**C**) Fabrication process of the membrane. (**D**) Chemical reactions in the post-modification and cross-linking steps. Reproduced with permission from Reference [172]. Copyright 2019 Royal Society of Chemistry.

Thus, 2D CS formation by using a linker leads to the assembly of a structure with high stability and quantum yield (QY). However, 2D CS formation decreases the solubility of NCs, which limits their processability and device applicability. Therefore, Zang et al. [172] recently established a method to polymerize 2D CS to overcome this problem and provide materials suitable for practical use (Figure 27A). In this study, Ag_12_(S-*^t^*Bu)_6_(CF_3_COO)_6_ NCs reported in their previous work (Figure 21) [168] were used as nodes. Moreover, 1,4-bis (pyrid-4-yl)benzenamine (bpz-NH_2_, Scheme 2(13)) was used as the linker. The amino group of the bpz-NH_2_ linker played an important role in polymerization. First, 2D CS crystals consisting of Ag_12_(S-*^t^*Bu)_6_(CF_3_COO)_6_ and bpz-NH_2_ were fabricated (Figure 27B). The crystal size was limited to about 200–300 nm by quenching the reaction within 1 min. A 2D CS film was obtained by reacting the crystals with methacrylic anhydride (MA). MA bound to the amino group of bpz-NH_2_ (Figure 27C) and was then polymerized with acrylate monomers butyl methacrylate (BMA) and triethylene glycol dimethacrylate (TEGDMA), as shown in Figure 27D.

**Figure 28:**
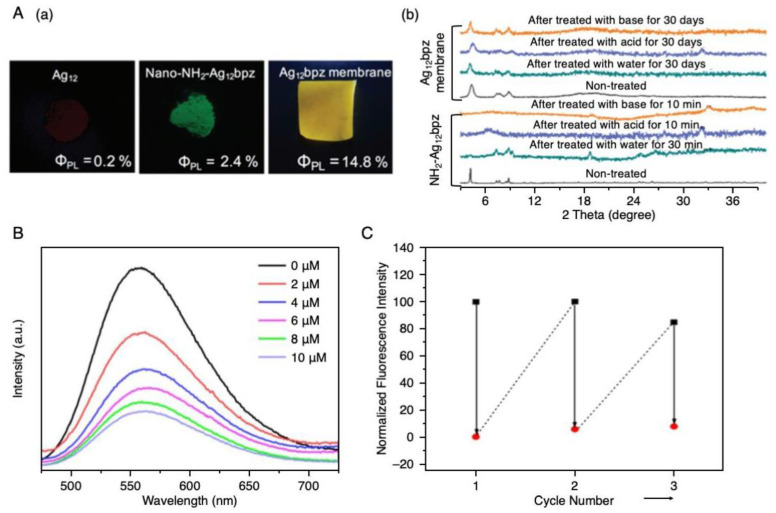
(**A**) (a) Photographs of the Ag_12_ clusters, nano-NH_2_-Ag_12_bpz, and an Ag_12_bpz membrane under 365 nm ultraviolet light irradiation. (b) PXRD patterns of NH_2_-Ag_12_bpz and the Ag_12_bpz membrane upon treatment with water, base, and acid for different periods. (**B**) Fluorescence spectra showing the response of the Ag_12_bpz membrane to the incremental addition of a nitrobenzene solution. (**C**) Cycling test of the Ag_12_bpz membrane upon exposure to nitrobenzene vapor. Reproduced with permission from Reference [172]. Copyright 2019 Royal Society of Chemistry.

The resulting membrane exhibited PL with a QY of 14.8%, which was higher than that of the unpolymerized 2D CS crystals (Figure 28A(a)). The increased PL intensity was ascribed to the polymerization strengthening the structure of the 2D CS, which suppressed molecular vibrations and thus nonradiative decay. The membrane was stable in water regardless of pH (Figure 28A(b)). The researchers also attempted to use the membrane to sense the harmful substance nitrobenzene in solution. The results revealed that the membrane was able to detect nitrobenzene with a sensitivity of 3.19 ppb (Figure 28B). This membrane also displayed high reusability (Figure 28C). These results indicate that polymerizing 2D CSs is an effective approach to obtain Ag NCs suitable for applications.

## 4. Three-Dimensional Structures

Ag NCs are often used as nodes in 3D CSs. Because 3D CSs generally possess stronger frameworks than those of 2D CSs, the formation of 3D CSs is effective to enhance the stability of Ag NCs and thereby improve their physical properties. In 3D CS formation, the principles of NC assembly are similar to those in 1D and 2D CS formation, although the ligands used are often different. In Section 4.1, Section 4.2 and Section 4.3, we focus on the assembly of 3D CSs via the formation of Ag−O, Ag−S, or Ag−Cl bonds (Figure 2B), control of counterions (Figure 2C), and the introduction of linker molecules (Figure 2D), respectively. The metal NCs, connection modes, linker molecules, year reported, and reference numbers for these studies are summarized in Table 3. Several of the ligands used in 3D CSs are shown in Scheme 1. The organic molecules used as linkers are illustrated in Scheme 2.

### 4.1. Connection via Ag−O, Ag−S, or Ag−Cl Bonds

**Figure 29:**
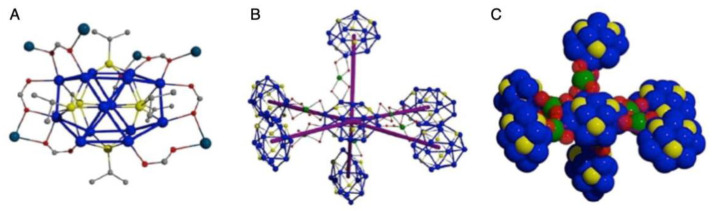
(**A**) Perspective view of Ag_14_(S-*i*Pr)_6_(CF_3_COO)_11_(H_2_O)_3_(CH_3_OH)Ag_3_. (**B**) Ball-and-stick and (**C**) space-filling diagrams showing the spatial arrangement of a central cluster surrounded by six adjacent clusters. Ag = blue, cyan, and green, S = yellow, O = red, C = gray. Reproduced with permission from Reference [148]. Copyright 2017 Wiley-VCH.

In 2017, Mak et al. [148] formed a 3D CS consisting of Ag_14_(S-*i*Pr)_6_(CF_3_COO)_11_(H_2_O)_3_(CH_3_OH) NCs (Figure 29A). The NCs were connected via O−Ag−O bonds formed between CF_3_COO^−^ and Ag ions (Figure 29B,C). Each Ag NC was connected to six other NCs, thereby forming a distorted octahedral-like coordination structure. It was speculated that this 3D CS formed by the assembly of NCs after NC generation.

**Figure 30:**
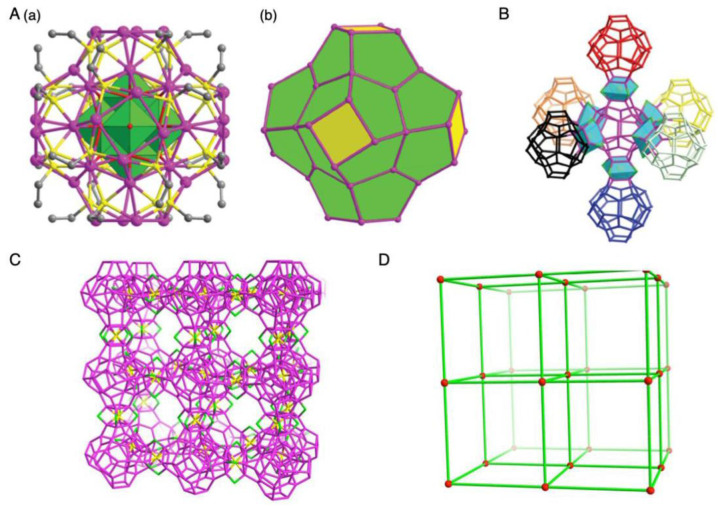
(**A**) Structure of (a) Ag_44_(Mo_6_O_19_)(S-Et)_24_(SCl_4_)_3_ and (b) the Mo_6_O_19_^2−^ anion template. Tetragons (yellow) and pentagons (green) in an Ag_44_ cage are shown. (**B**) Connections (cyan polyhedra) between Ag_44_ subunits (highlighted in different colors) in the 3D framework. (**C**) Framework and (**D**) simplified primitive cubic topology with the Ag_44_ subunit as a node (represented as red balls) of the Ag_44_(Mo_6_O_19_)(S-Et)_24_(SCl_4_)_3_ 3D CS. Reproduced with permission from Reference [149]. Copyright 2019 Royal Society of Chemistry.

In 2019, Sun and colleagues also produced a 3D CS, and theirs consisted of Ag_44_(Mo_6_O_19_)(S-Et)_24_(SCl_4_)_3_ NCs (Figure 30A) containing a POM as an anion template (Figure 30B); these NCs were reported in their paper on 1D CS (Figure 9) and 2D CS (Figure 19) formation [149]. In this 3D CS, Mo_6_O_19_^2−^ was used as an anion template, which was different from the case of the 1D and 2D CSs, in which the POM V_10_O_28_^6−^ was located in the center of the cluster. This was the first report in which Mo_6_O_19_^2−^ was used as an anion template of Ag NCs. Mo_6_O_19_^2−^ has octahedral symmetry, and thereby the outer Ag_44_(S-Et)_24_ layer also displayed high symmetry (Figure 30C). The Ag_44_ shell had six quadrangles and 24 pentagonal faces. Ag at the vertices of these six quadrangles was connected with one S atom and four Cl atoms, leading to the formation of a 3D CS consisting of Ag_44_(Mo_6_O_19_)(S-Et)_24_(SCl_4_)_3_ (Figure 30D). The S and Cl atoms used in the connections were generated by the decomposition of S-Et and CH_2_Cl_2_ during the CS synthesis.

**Figure 31:**
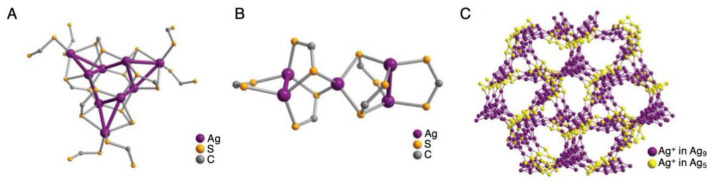
(**A**) Structures of the Ag_9_ cluster, (**B**) Ag_5_ subunits, and (**C**) 3D framework of Ag_17_(C_5_NS_2_H_10_)_14_. Reproduced with permission from Reference [173]. Copyright 2019 Royal Society of Chemistry.

The 3D CS in which Ag NCs are linked by dithiocarb, reported in 2019 by Gao et al. [173], should also be included in this category. The researchers first synthesized an Ag_11_S(C_5_NS_2_H_10_)_9_ precursor (C_5_NS_2_H_10_ = diethyldithiocarbamate, Scheme 1(20)) [174]. The obtained precursor was reacted under high pressure, in an autoclave, to form a 3D CS with Ag_17_(C_5_NS_2_H_10_)_14_ as a repeating unit. This structure consists of Ag_9_ NCs bound to twelve C_5_NS_2_H_10_ (Figure 31A) and Ag_5_ NCs containing six C_5_NS_2_H_10_ (Figure 31B). The 3D CS (Figure 31C) was formed by the sharing of S between the two types of NCs. In these structures, the Ag_9_ NCs were the nodes for three-point bridges and the Ag_5_ NCs were the nodes for four-point bridges.

### 4.2. Control of Counterions

**Figure 32:**
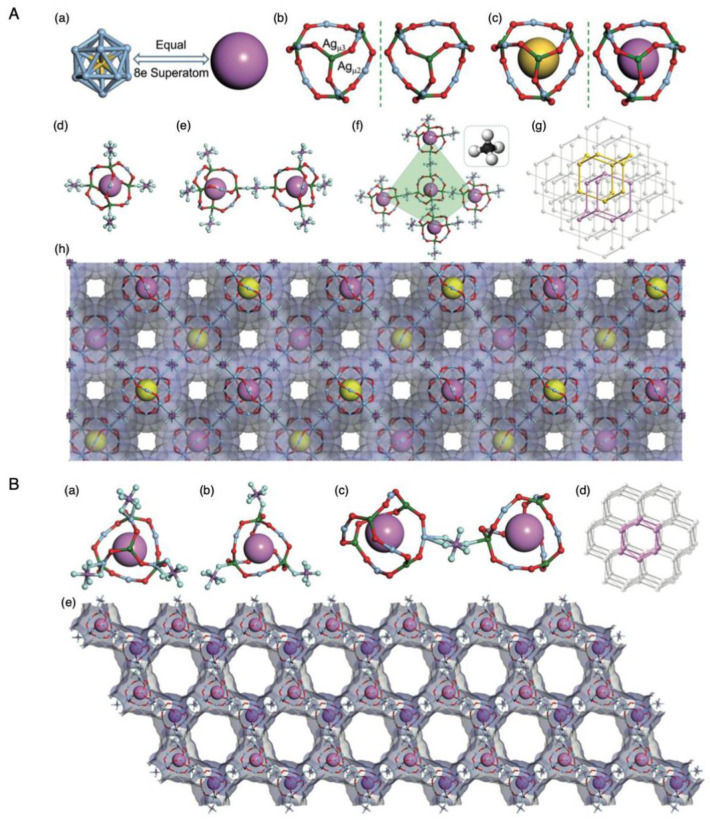
(**A**) Structure of the Au_1_Ag_22_ superatom complex and interpenetrating 3D channel framework assembled from alloy NC monomers (for clarity, C and H atoms are omitted). (a) Icosahedral Au_1_Ag_12_ core, (b) cage-like Ag_10_(SR)_12_ complex shell, (c) a pair of Au_1_Ag_22_ isomers, (d) the connection of SbF_6_^−^ and alloy NCs, (e) two alloy NCs connected by SbF_6_^−^, (f) tetrahedral structure of NC monomers (the inset shows methane), (g) topology of the diamond-like structure, and (h) interconnected channels of Au_1_Ag_22_ along the *z*-axis. The left- and right-handed enantiomers in (c), (g), and (h) are highlighted in pink and yellow, respectively. Atoms are denoted in conventional colors: Au = gold, Ag in core and the Agμ2 motif = pale blue, Ag in the Agμ3 motif = green, S = red, F = light turquoise, Sb = purple. (**B**) Crystal and channel structure of left-handed chiral 3D channel framework (C and H atoms are omitted for clarity). (a) The connection of Ag and SbF_6_^−^, (b) the connection of Ag and SbF_6_^−^, (c) two alloy NCs linked by SbF_6_^−^, (d) illustration of the hexagonal network structure, and (e) schematic of the large hexagonal channel structure. Note that the packing pattern of the right-handed chiral 3D channel framework was the same as that of the left-handed chiral 3D channel framework. Reproduced with permission from Reference [175]. Copyright 2020 Wiley-VCH.

Very recently, Zhu’s group synthesized two types of 3D CSs in which [Au_1_Ag_22_(S-Adm)_12_]^3+^ NCs (S-Adm = 1-adamantanethiolate, Scheme 1(21)) were connected in three dimensions via hexafluoroantimonate ions (SbF_6_^−^) [175]. The [Au_1_Ag_22_(S-Adm)_12_]^3+^ node had a geometric structure in which an icosahedral Au_1_Ag_12_ alloy core (Figure 32A(a)) was surrounded by an oligomer with a chemical composition of Ag_10_(S-Adm)_12_ (Figure 32A(b),(c)). The [Au_1_Ag_22_(S-Adm)_12_]^3+^ NCs formed as a pair of optical isomers, depending on the winding method of the oligomer (Figure 32A(b),(c)). In the first 3D CS, [Au_1_Ag_22_(S-Adm)_12_](SbF_6_)_2_Cl was a structural unit, and two SbF_6_^−^ were connected to [Au_1_Ag_22_(S-Adm)_12_]^3+^ via an Ag−F−Ag bond, to form the 3D CS (Figure 32A(d),(e)). This 3D CS possessed a diamondlike structure (Figure 32A(f)) consisting of interpenetrating clockwise and counterclockwise optical isomers (Figure 32A(g)), which led to a small pore diameter of 6.2 Å (Figure 32A(h)). In the second 3D CS, [Au_1_Ag_22_(S-Adm)_12_](SbF_6_)_3_ was a structural unit, and the 3D CS was formed by connecting [Au_1_Ag_22_(S-Adm)_12_]^3+^ to three SbF_6_^−^ via Ag−F−Ag bonds (Figure 32B(a)–(c)). This structure only contained clockwise or counterclockwise optical isomers (Figure 32B(d)). As a result, this 3D CS had a larger pore diameter (15 Å, Figure 32B(e)) than that of the first 3D CS (6.2 Å, Figure 32A).

**Figure 33:**
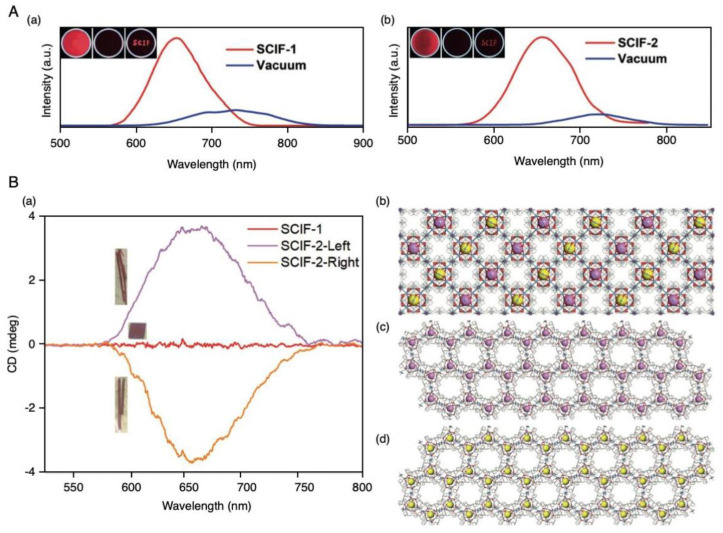
(**A**) Luminescence switching response to protic solvents. Fluorescence of films coated with (a) the interpenetrating 3D CS (SCIF-1) and (b) the left-handed 3D CS (SCIF-2) before and after solvent evaporation. Inset images show solvatochromic photographs of the corresponding films excited with 365 nm ultraviolet light before (left) and after desolvation (middle), and fluorescence recovery after treatment with *n*-hexane containing 5% ethanol (right). (**B**) CPL spectra of SCIF-1, SCIF-2 (left-handed), and SCIF-2 (right-handed) single crystals and the superstructures of these three crystal samples. (a) CPL spectra of SCIF-1, SCIF-2 (left-handed), and SCIF-2 (right-handed). Insets show photographs of the corresponding crystals. (b) Crystal structure of the SCIF-1 framework. (c) Crystal structure of the SCIF-2 (left-handed framework). (d) Crystal structure of the SCIF-2 (right-handed) framework. Reproduced with permission from Reference [175]. Copyright 2020 Wiley-VCH.

Study of the physical and chemical properties of the 3D CSs revealed that both exhibited red PL in the presence of polar solvents such as CH_3_OH, ethanol, and water, which disappeared when the solvent was evaporated (Figure 33A). This behavior indicates that the obtained 3D CSs can function as sensors for polar solvents. The 3D CS composed of only the right- or left-handed enantiomer exhibited circularly polarized luminescence (CPL) (Figure 33B).

### 4.3. Introduction of Linker Molecules

The formation of 3D CSs by using linker molecules is a technique often used to fabricate molecular assemblies and MOFs. When a 3D CS composed of metal NCs is formed by such a method, in addition to increasing the stability of the NCs, it is also expected to adsorb gas molecules within its pores and behave as a catalyst with high selectivity because of the narrow pores. Furthermore, because the metal NCs, which are used as nodes, have more diversity in terms of coordination direction than that of metal ions, metal NC-based MOFs may have different connection modes from those of normal MOFs formed by using metal ions as nodes, and thereby they construct novel framework structures. Thus, metal NC-based MOFs possess not only the characteristics of individual metal NCs and MOFs, but also the possibility to produce new functions through synergistic effects.

**Figure 34:**
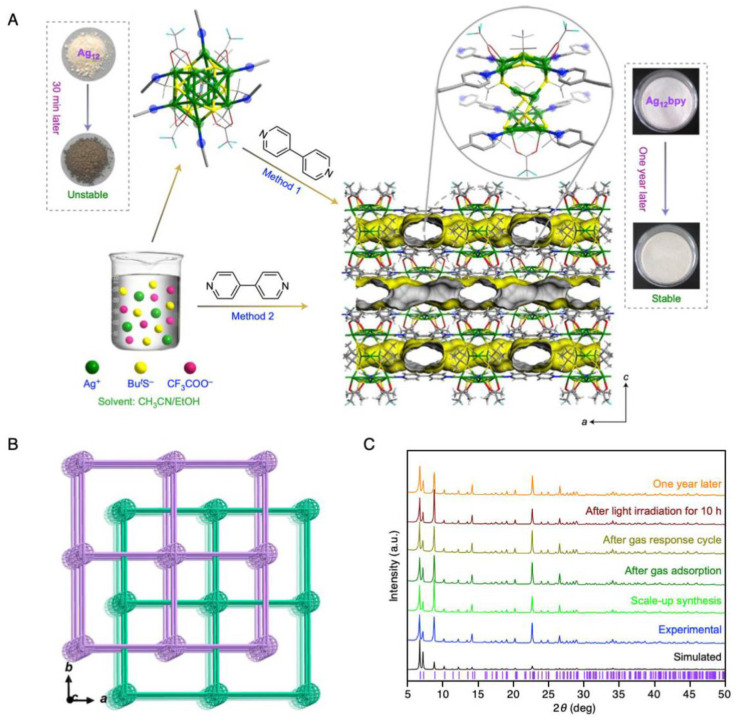
(**A**) Schematic representation of the ligand-exchange strategy used to obtain Ag_12_(S-*^t^*Bu)_8_(CF_3_COO)_4_(bpy)_4_ crystals (Method 1, giving low yield) and one-pot synthesis (Method 2, for gram-quantity production) under identical conditions. Interconnected channels of Ag_12_(S-*^t^*Bu)_8_(CF_3_COO)_4_(bpy)_4_ viewed along the *a* and *b* axes, where the yellow surface represents the pore surface. Ag = green, C = gray, O = red, S = yellow, F = turquoise. H atoms are omitted for clarity. Inset are photographs showing the changes of Ag_12_(S-*^t^*Bu)_6_(CF_3_COO)_6_(CH_3_CN)_6_ and Ag_12_(S-*^t^*Bu)_8_(CF_3_COO)_4_(bpy)_4_ crystals under ambient conditions. (**B**) Schematic representation of the topology of Ag_12_(S-*^t^*Bu)_8_(CF_3_COO)_4_(bpy)_4_ along the *c*-axis. The square windows in one double layer are blocked by Ag–S cluster nodes of adjacent layers. (**C**) PXRD patterns of Ag_12_(S-*^t^*Bu)_8_(CF_3_COO)_4_(bpy)_4_ (in ascending order): simulated, experimental, gram-scale synthesis, after gas adsorption experiments (O_2_, N_2_, ethanol), after sensing experiments (O_2_/N_2_, O_2_/vacuum, ethanol/air), after 10 h of visible-light irradiation under a Xe lamp equipped with a 420 nm cutoff filter, and the sample vial after one year under ambient conditions. Reproduced with permission from Reference [168]. Copyright 2017 Springer-Nature.

Zang’s group have been energetically researching 3D CSs with linkers, as well as the cases of 1D and 2D CSs. First, in 2017, Zang et al. [168] formed an Ag_12_ NC-based MOF (Ag_12_(S-*^t^*Bu)_8_(CF_3_COO)_4_(bpy)_4_) in which Ag_12_(S-*^t^*Bu)_8_(CF_3_COO)_4_ was bridged by bpy (Figure 34A). The obtained Ag_12_ NC-based MOF possessed a bilayer structure (Figure 34B). The formation of such a 3D CS markedly improved the stability of the Ag_12_ NCs. For example, a crystal of the individual Ag_12_(S-*^t^*Bu)_6_(CF_3_COO)_6_(CH_3_CN)_6_ NCs discolored in just 30 min when left in the atmosphere. In contrast, the Ag_12_ NC-based MOF showed almost no change in crystallinity, even when left in the air for one year (Figure 34C). The 3D CS also showed high stability during long-term gas adsorption and irradiation with visible light for several hours.

**Figure 35:**
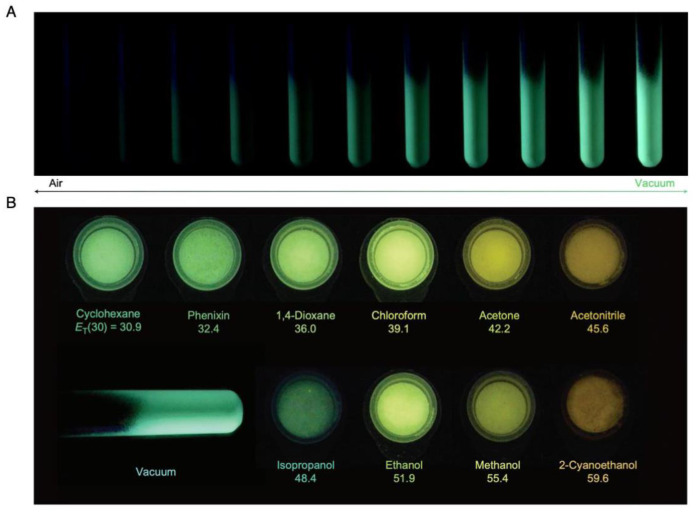
(**A**) Photographs of Ag_12_(S-*^t^*Bu)_8_(CF_3_COO)_4_(bpy)_4_ excited by 365 nm light in a glass tube, beginning under vacuum and then filling with air (from left to right). (**B**) Photographs of the luminescence responses of Ag_12_(S-*^t^*Bu)_8_(CF_3_COO)_4_(bpy)_4_ to different VOCs under 365 nm ultraviolet light irradiation. Reproduced with permission from Reference [168]. Copyright 2017 Springer-Nature.

The formation of the 3D CS greatly changed the PL properties of the NCs. The individual Ag_12_ NCs exhibited red PL with low QY. Conversely, the Ag_12_ NC-based MOF exhibited green PL under vacuum, which was quenched by O_2_ in the atmosphere (Figure 35A). The PL emission wavelength of the 3D CS under vacuum was independent of temperature and excitation wavelength, and its QY was 60 times higher than that of individual Ag_12_ NCs. The authors ascribed this high QY to the efficient suppression of nonradiative decay in the 3D CS. Moreover, the fact that the PL of the 3D CS is quenched by O_2_ means that it is highly sensitive to O_2_. The 3D CS showed a fast response to O_2_ in experiments in which the atmosphere was repeatedly switched between air and N_2_. No such O_2_ response was observed for the individual Ag_12_ NCs. Based on these results, they suggested that the Ag_12_ NC-based MOF can be applied as an O_2_ sensor. In addition, the Ag_12_ NC-based MOF was able to adsorb volatile organic compounds (VOCs) in its pores. The VOC-containing Ag_12_ NC-based MOF exhibited different PL colors, depending on the kind of VOC (Figure 35B). This indicates that the Ag_12_ NC-based MOF displays solvatochromism and therefore can be used for VOC detection.

**Figure 36:**
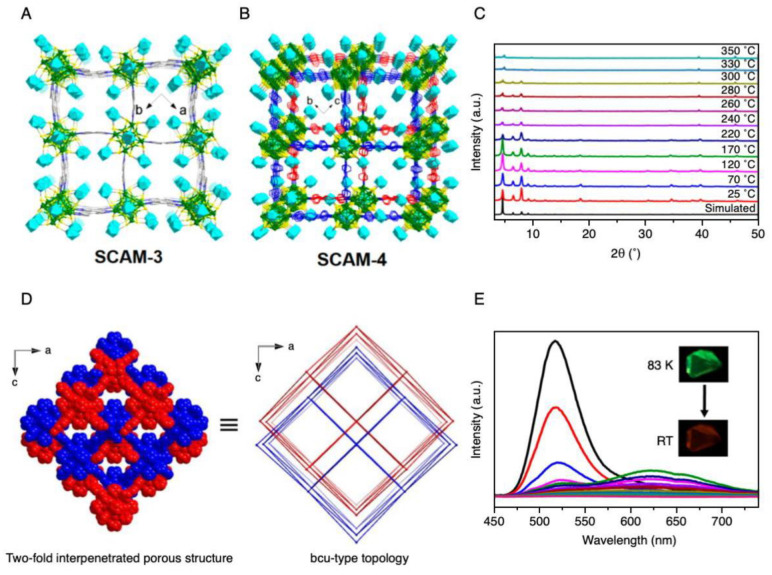
Three-dimensional CSs composed of (**A**) Ag_14_(DT-*o*-C)_6_ NCs and bpy (SCAM-3) and (**B**) Ag_14_(DT-*o*-C)_6_ NCs and 1,4-bis(4-pyridyl)benzene (SCAM-4). (**C**) Variable-temperature PXRD of SCAM-4 from 25 to 350 °C. (**D**) Different view of SCAM-4. (**E**) Evacuated SCAM-4 (excited at 380 nm) from −190 to 25 °C in air. Inset are corresponding photographs of SCAM-4 under 365 nm ultraviolet light irradiation. Reproduced with permission from Reference [156]. Copyright 2018 American Chemical Society.

In a paper on Ag_14_(DT-*o*-C)_6_ NCs published in 2018 (Figure 13 and Figure 22), the same group reported that a 3D CS containing Ag_14_(DT-*o*-C)_6_ NCs formed when bpy was used as a linker (Figure 36A) [156]. The Ag_14_ NC nodes in the 3D CS had a face-centered cubic structure like that of other Ag_14_ NCs. This 3D CS was formed by connecting the eight vertices of each Ag_14_ NC with bpy linker molecules. However, this 3D CS was not stable after solvent evaporation, similar to the case of the corresponding 1D CS (Figure 13) and 2D CS (Figure 22) [156]. Therefore, they synthesized a 3D CS with an interpenetrating framework by using 1,4-bis(4-pyridyl)benzene (Scheme 2(14)) as a linker, in order to decrease the pore size and form a 3D CS with a strong framework (Figure 36B). The obtained 3D CS showed high thermal stability, remaining intact up to 220 °C (Figure 36C), and possessed pores with a diameter of about 1.12 nm (Figure 36D). This 3D CS showed optical absorption over a wide wavelength range and thermochromism (Figure 36E).

**Figure 37:**
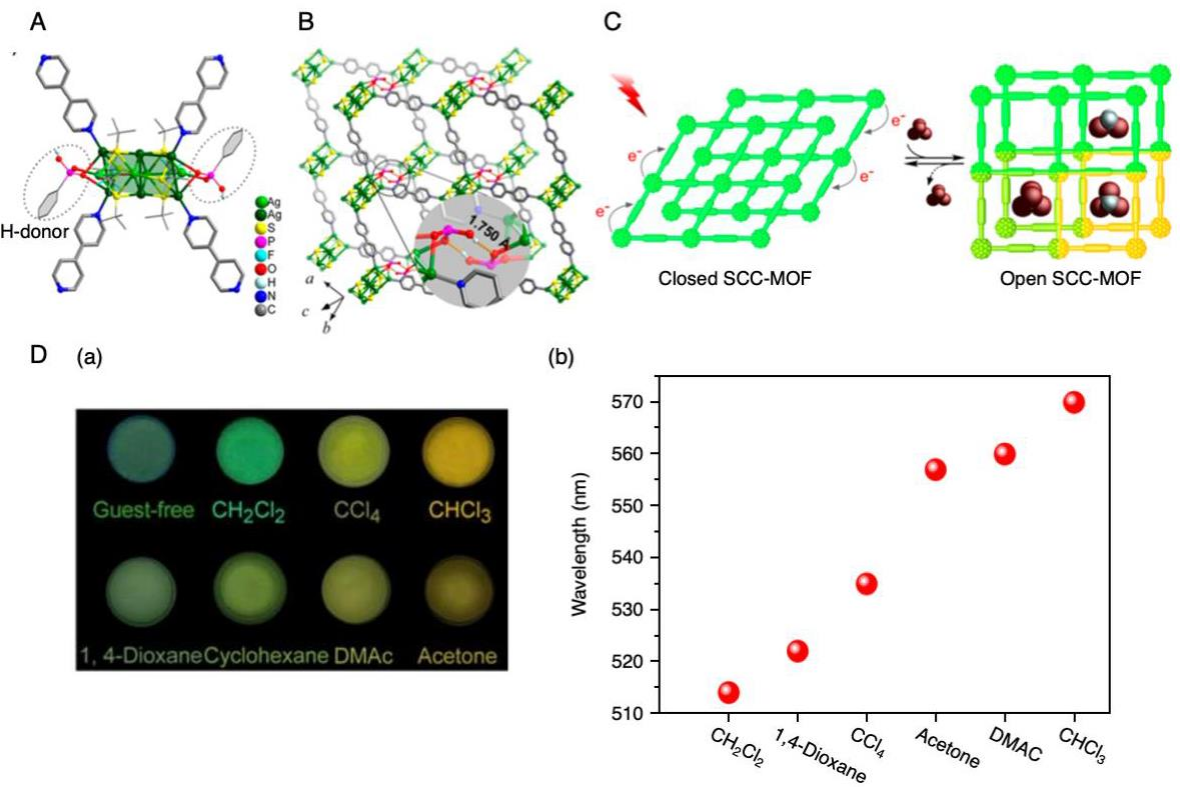
(**A**) Perspective view of the coordination environment of the Ag_10_(S-*^t^*Bu)_6_ core in Ag_10_(S-*^t^*Bu)_6_(CF_3_COO)_2_(PhPO_3_H)_2_(bpy)_2_ at −173 °C. (**B**) Two-layer stack of the host framework of Ag_10_(S-*^t^*Bu)_6_(CF_3_COO)_2_(PhPO_3_H)_2_(bpy)_2_ with complementary hydrogen bonding (O−H···O; the H···O distance is 1.750 Å) between interlayer −PO_2_OH moieties. (**C**) Illustration of reversible pore open/closed structural transformation induced by CH_2_Cl_2_, CHCl_3_, and CCl_4_ (represented as space-filling models) and switchable solvatochromism. (**D**) (a) Luminescence images of Ag_10_(S-*^t^*Bu)_6_(CF_3_COO)_2_(PhPO_3_H)_2_(bpy)_2_/solvent (guest free, CH_2_Cl_2_, CHCl_3_, CCl_4_, 1,4-dioxane, cyclohexane, DMAC, and acetone) combinations under 365 nm ultraviolet light irradiation. (b) Emission maxima of various Ag_10_(S-*^t^*Bu)_6_(CF_3_COO)_2_(PhPO_3_H)_2_(bpy)_2_/solvent combinations at room temperature. Reproduced with permission from Reference [176]. Copyright 2018 American Chemical Society.

**Figure 38:**
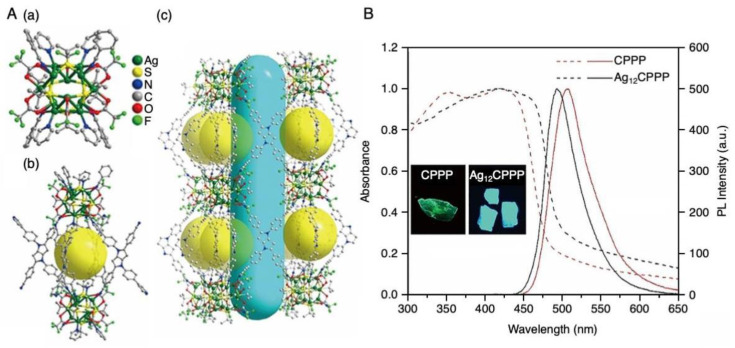
(**A**) Structures of (a) Ag chalcogenolate cluster nodes, (b) cage in Ag_12_(S-*^t^*Bu)_6_(CF_3_COO)_6_(CPPP)_2_ (Ag_12_CPPP), and (c) distribution of the cages in Ag_12_CPPP. All H atoms and guest solvent molecules are omitted for clarity. (**B**) Solid-state absorption (dashed lines) and emission (solid lines) spectra of Ag_12_CPPP and CPPP at room temperature. Inset are photographs of the crystals of CPPP and Ag_12_CPPP under 365 nm ultraviolet light irradiation. Reproduced with permission from Reference [177]. Copyright 2019 Wiley-VCH.

Zang et al. [176] have also produced several other functional Ag NC-based MOFs. For example, in 2018, they reported the synthesis of a flexible Ag NC-based MOF. This structure consisted of 2D layers of Ag_10_(S-*^t^*Bu)_6_(CF_3_COO)_2_(PhPO_3_H)_2_ NCs linked via bpy, which were stacked through hydrogen bond (O-H···O) and C-H···O interactions, to form the 3D CS (Figure 37A,B). The 2D CS layers were thus linked by weak interactions, which facilitated the sliding of the layers, allowing the 3D CS to undergo structural deformation in response to guest organic molecules (Figure 37C). This Ag_10_ NC-based MOF exhibited green PL in air. Upon inclusion of guest organic molecules, it exhibited PL with an emission color depending on the guest organic molecule (Figure 37D). As such, this Ag_10_ NC-based MOF has potential as a sensor for distinguishing VOCs by its PL color. In 2019, they also formed a 3D CS consisting of Ag_12_(S-*^t^*Bu)_6_(CF_3_COO)_6_ NC nodes and 2,5-bis(4-cyanophenyl)-1,4-bis(4-(pyridine-4-yl)-phenyl)-1,4-dihydropyrrolo[3,2-b]pyrrole (CPPP, Scheme 2(15)) as a linker (Figure 38A) [177]. This was the first report in which a nitrile group (-CN) was used to link Ag NCs. The obtained 3D CS exhibited PL with a higher QY (61%) than that of CPPP in solution and solid states because the aggregation-induced quenching of CPPP was suppressed in the 3D CS (Figure 38B).

**Figure 39:**
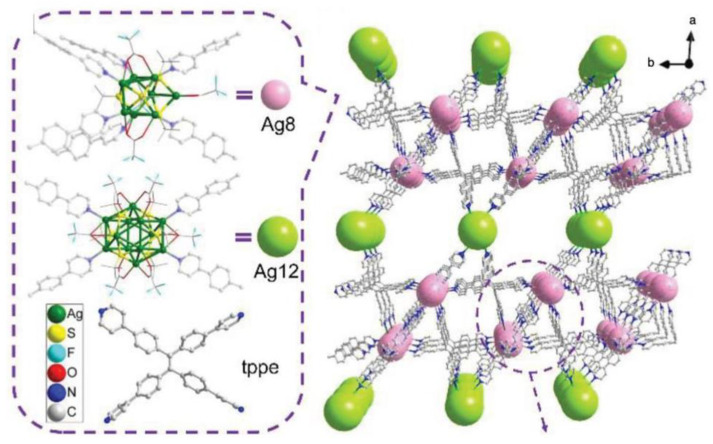
Structure of the Ag_8_ cluster, Ag_12_ cluster, and tppe ligand, and single net of the [Ag_12_(S-*^t^*Bu)_6_(CF_3_COO)_6_]_0.5_[Ag_8_(S-*^t^*Bu)_4_(CF_3_COO)_4_](tppe)_2_(DMAC)_10_ framework viewed along the *c*-axis. DMAC molecules are omitted for clarity. Reproduced with permission from Reference [178]. Copyright 2019 Wiley-VCH.

**Figure 40:**
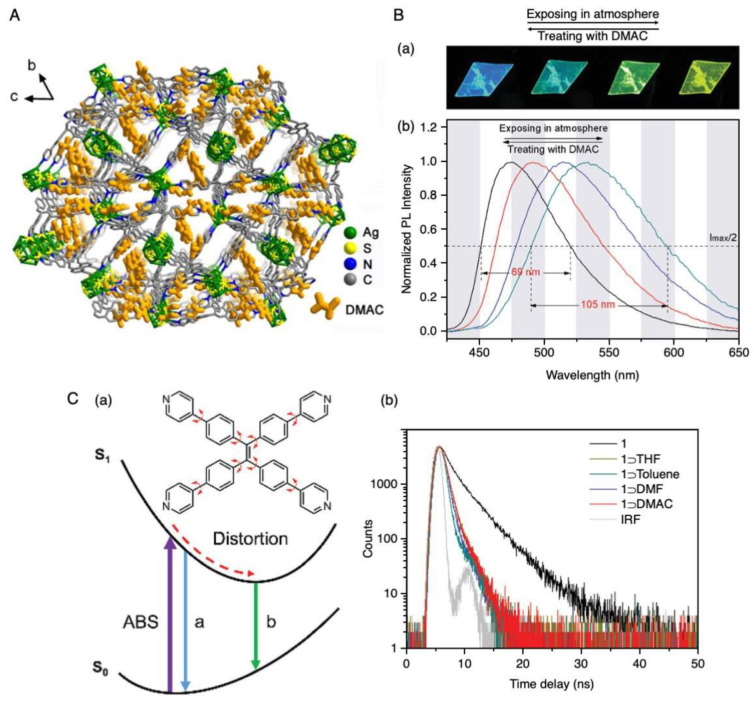
(**A**) Distribution of DMAC molecules in the [Ag_12_(S-*^t^*Bu)_6_(CF_3_COO)_6_]_0.5_[Ag_8_(S-*^t^*Bu)_4_(CF_3_COO)_4_](tppe)_2_(DMAC)_10_ framework. (**B**) (a) Gradual fluorescence changes of the same [Ag_12_(S-*^t^*Bu)_6_(CF_3_COO)_6_]_0.5_[Ag_8_(S-*^t^*Bu)_4_(CF_3_COO)_4_](tppe)_2_(DMAC)_10_ crystal under atmospheric exposure and (b) normalized fluorescence spectra of [Ag_12_(S-*^t^*Bu)_6_(CF_3_COO)_6_]_0.5_[Ag_8_(S-*^t^*Bu)_4_(CF_3_COO)_4_](tppe)_2_(DMAC)_10_. (**C**) (a) Proposed fluorescence decay paths in [Ag_12_(S-*^t^*Bu)_6_(CF_3_COO)_6_]_0.5_[Ag_8_(S-*^t^*Bu)_4_(CF_3_COO)_4_](tppe)_2_(DMAC)_10_ (path a) and [Ag_12_(S-*^t^*Bu)_6_(CF_3_COO)_6_]_0.5_[Ag_8_(S-*^t^*Bu)_4_(CF_3_COO)_4_](tppe)_2_ (**1**) (path b) and (b) fluorescence decay profiles of [Ag_12_(S-*^t^*Bu)_6_(CF_3_COO)_6_]_0.5_[Ag_8_(S-*^t^*Bu)_4_(CF_3_COO)_4_](tppe)_2_ in DMAC, THF, toluene, and DMF (denoted as **1**⊃DMAC, **1**⊃THF, **1**⊃Toluene, and **1**⊃DMF, respectively). Reproduced with permission from Reference [178]. Copyright 2019 Wiley-VCH.

In the above 3D CSs, the nodes consisted of only one kind of Ag NCs. Tang et al. [178] synthesized an Ag NC-based MOF that had two types of Ag NCs as nodes. This Ag NC-based MOF was composed of 1,1,2,2-tetrakis(4-(pyridin-4-yl)phenyl)-ethene (tppe, Scheme 2(16)), Ag_12_(S-*^t^*Bu)_6_(CF_3_COO)_6_, and Ag_8_(S-*^t^*Bu)_4_(CF_3_COO)_4_ (Figure 39). In the 3D CS, one Ag_12_(S-*^t^*Bu)_6_(CF_3_COO)_6_ NC and three Ag_8_(S-*^t^*Bu)_4_(CF_3_COO)_4_ NCs were bound to the four N atoms of tppe. The estimated pore volume of this Ag NC-based MOF was 40.9%. DMAC, which was used as a solvent in the synthesis, was present in the pores of the 3D CS immediately after synthesis (Figure 40A). When the obtained 3D CS was exposed to the atmosphere, DMAC was removed, while maintaining the framework of the 3D CS. The Ag NC-based MOF thus obtained exhibited PL in the visible region because tppe is a light-emitting molecule. The PL wavelength of the 3D CS depended on the presence or absence of DMAC in its pores (Figure 40B). When DMAC was present in the pores of the framework, intramolecular rotation of tppe was suppressed, which changed the excited-state dynamics of the 3D CS. The change in the emission behavior of the Ag NC-based MOF induced by DMAC was ascribed to this change of its excited-state characteristics (Figure 40C).

**Figure 41:**
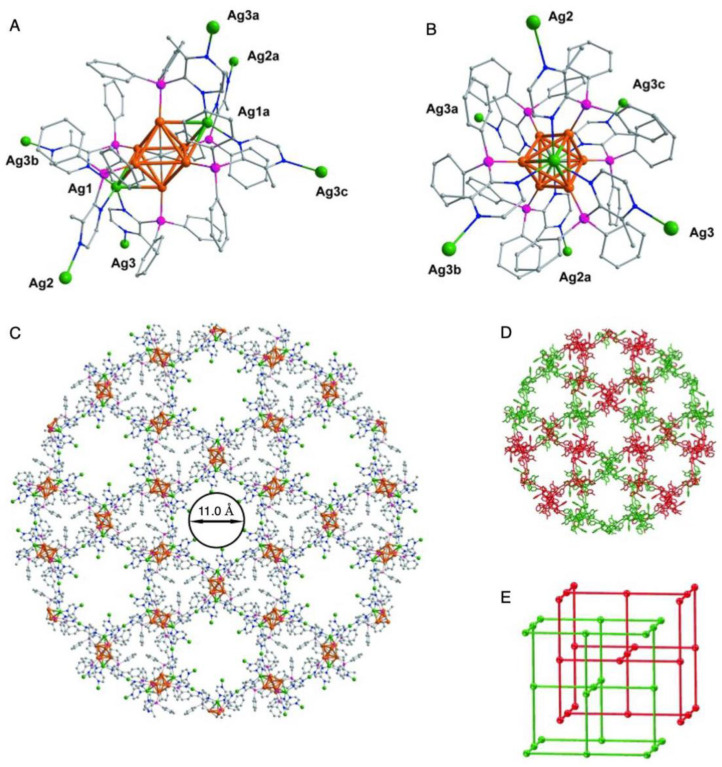
(**A**) Structure of [C(Au-mdppz)_6_](BF_4_)_2_. (**B**) Four extensions: Ag3, Ag3a, Ag3b, and Ag3c. Note that Ag2 and Ag2a are not involved in the structural extension. (**C**) Perspective view of the 3D CS along the *c* direction. Au = orange, Ag = green, P = purple, N = blue, C = gray. (**D**) Two interpenetrating nets shown in different colors; anions and solvent molecules are omitted for clarity. (**E**) Schematic representation of NbO topology in the 3D CS. Reproduced with permission from Reference [179]. Copyright 2014 Wiley-VCH.

Wang and colleagues reported the formation of a 3D CS, using metal NCs as linkers, before the use of metal NCs as nodes was developed [179]. In 2014, they succeeded in forming an NbO-type MOF by using Ag ions as nodes and [C(Au-mdppz)_6_](BF_4_)_2_] (mdppz = 2-(3-methylpyrazinyl)diphenylphosphine, Scheme 2(17)) NCs as linkers (Figure 41). [C(Au-mdppz)_6_](BF_4_)_2_ has a framework with C in the center (Figure 41A) and is luminescent. The 3D CS was formed by the outer N atom of mdppz (Scheme 2(17)) binding to an Ag ion (Figure 41B,C). The obtained 3D CS consisted of two interpenetrating frameworks (Figure 41D,E) with a 1D channel in the *c*-axis direction (Figure 41C). Because a luminescent NC was used as the linker, the obtained MOF also showed green PL. The 3D CS displayed a PL QY of 25.6%, which was much higher than that of the luminescent NCs (1.5%). This increase of QY was caused by the strengthening of the framework of the linker NCs upon MOF formation and the excited-state perturbation induced by the coordination of Ag ions.

## 5. Summary

In this review, representative studies on the formation of 1D, 2D, and 3D CSs in which metal NCs were self-organized and regularly linked were summarized. From this summary, the following points became clear.

(1) **Methods.** The methods to connect metal NCs that have been reported to date can be roughly divided into the following five categories: (i) direct connection by formation of metal−metal bonds (Figure 2A); (ii) connection by Ag−O, Ag−S, or Ag−Cl bond formation (Figure 2B); (iii) connection by counterions (Figure 2C); (iv) connection by linker molecules (Figure 2D); and (v) connection by inter-ligand interactions (Figure 2E; not introduced in this review).
(2) **Diversity.** Among CSs produced by the above methods, there are many examples of the formation of 1D, 2D, and 3D CSs through the use of methods (ii) and (iv). An important point when constructing CSs by these methods is the design of the ligand of the NCs and linker, respectively. It is presumed that the control of these species is relatively easy, which has led to the wider utilization of methods (ii) and (iv) than of the other methods. In particular, for method (iv), existing knowledge obtained in the study of normal MOFs can be considered.
(3) **Metal element.** To directly connect metal NCs, it is effective to use Au as a main element because it forms strong aurophilic interactions (intermetal interactions). In the connections involving metal−O or metal−Cl bonds, it is effective to use Ag as a main element because it readily bonds with O or Cl. Moreover, in the connections using bpy as a linker, Ag is attractive as the main element because of the high affinity of N and Ag.
(4) **Stability.** The formation of a CS generally improves the thermal stability of the component metal NCs regardless of the connection mode.
(5) **Electronic structure.** The formation of a CS often causes the band gap of the NC to narrow. This means that CS formation allows the use of a broader wavelength range of light, opening up the possibility of visible-light-driven photocatalysis by using CSs.
(6) **PL properties.** For 2D and 3D CS using linkers, CS formation often leads to an increase in PL emission intensity. When metal NCs are the PL source, there are many cases in which dual emission peaks appear upon connection with a linker. In addition, the PL color of a CS often changes depending on the kind of VOC trapped in its pores.
(7) **Electrical conduction.** The electron conductivity of CSs changes dramatically depending on the distance between each metal NC and the mode of connection; 1D CS formed by the direct connection via metal−metal bond shows the higher conductivity than 1D CS connected through counter ion.
(8) **Possible applications.** The reported CSs have potential applications in fields such as electronic devices, luminescent devices, gas and temperature sensing, and photocatalysis.

This review allowed us to obtain a common understanding of the CSs reported to date and their functions. We hope that the knowledge thus clarified will lead to clear design guidelines for developing new CSs with desired functions in the future.

## 6. Outlook

It is expected that the following studies will be conducted in the future, leading to new CSs.

(1) **Use of other metal elements.** At present, mostly Au and Ag are used as the metal element in CSs. This is largely related to the high stability of Au and Ag NCs. For Ag NCs, the good connectivity between Ag and linker molecules is also related to this fact. On the other hand, several syntheses of individual copper (Cu) NCs have been reported recently [10,180,181,182,183]. In addition, other metal ions are often used in normal MOFs with metal ions as nodes [153]. CS formation of NCs based on Cu or other metals may also lead to materials with high thermal stability. In the future, it is expected that many elements will be used in CSs, thereby realizing various functions and decreased cost of such materials.
(2) **Use of the alloying effect.** At present, there are few examples in which alloy NCs are connected to form CSs [142,143,146]. Mixing different elements leads to NCs with physical/chemical properties and functions that are different from those of monometal NCs. In fact, for individual metal NCs, many cases have been reported in which new physical properties/functions appeared because of mixing/synergistic effects [66,184,185,186,187,188,189,190,191,192,193,194]. The previous studies have established basic techniques for the formation of CSs consisting of Ag NCs. In the future, it is expected that more functional materials will be created by extending such CS formation techniques to Ag-based alloy NCs.
(3) **Connection of reported metal NCs.** Ag NC-based MOFs are interesting because they can be synthesized by a one-pot process. However, in CSs formed by such a method, metal NCs that are stable only in the CS are often found as nodes. For individual metal NCs, many NCs have already been synthesized with atomic precision [1,2,3,4,5,6,7,8,9,10,11,12,13,14,15,16,17,18,19,20,21,22,23,24,25,26,27,28,29,30,31,32,33,34,35,36,37,38,39,40,41,42,43,44,45,46,47,48,49,50,51,52,53,54,55,56,57,58,59,60,61,62,63,64,65]. In addition, much information has been obtained on methods to generate novel functions in such NCs, including alloying [66,67,68]. In the future, it is expected that a method to more effectively utilize the reported metal NCs in CSs will be found. To achieve this, it may be necessary to establish new connection methods different from those described in this review (Figure 2).
(4) **Elucidation of electronic conductivity.** We believe that 1D CSs may be applied as nanodevices. However, at present, few experiments on the conductivity of 1D CSs have been reported [146,152]. In the future, it is expected that the conductivity of 1D CSs will be measured as a basic physical property. It is anticipated that the accumulation of such information will eventually lead to the production of nanodevices based on 1D CSs of metal NCs.
(5) **Exploration of other possible applications of connected structures.** Various applications, such as gas storage, gas separation, gas conversion, and reaction-selective catalysis, have been studied for normal MOFs with metal ions as nodes [117]. It has also been reported that, in the case of self-assembled complexes, a reaction different from that in the case of using an ordinary flask proceeds in the cage structure (i.e., the cage behaves as a nanoflask) [116]. In the future, it is expected that these possibilities will be investigated for metal NC-based MOFs and that their functions will be much higher than those of conventional MOFs and self-assembled complexes.

As mentioned in Section 1, the advance of bottom-up technology is essential for the further development of nanotechnology in the future. In previous research, multiple techniques to generate metal atoms on the molar scale in a solution and to self-organize them to form nanomaterials with the same number of constituent atoms and the same number of molecules have been studied; that is, precise synthesis techniques of metal NCs have been developed. However, to apply these metal NCs as devices and next-generation materials, developing techniques to assemble metal NCs to a size that is easy to handle is necessary (Figure 1). We hope that technologies that allow the self-organization of regularly arranged CSs composed of metal NCs will be further developed in the future. Ultimately, such nanotechnology is expected to enable resource conservation, energy conservation, decreased waste and environmental load, and the better use of time by human society.

## Figures and Tables

**Table 1:** Connection modes, NCs, linkers, publication years, and references for 1D CS.

Connection Mode	NC	Linker	Year	Ref.
Formation of metal−metal bond (Figure 2A)	[Au_25_(S-Bu)_18_]^0^	-	2014	[122]
	[Au_25_(S-Pen)_18_]^0^	-	2017	[140]
	[Au_24_Hg(S-Bu)_18_]^0^	-	2019	[141]
	[Au_24_Cd(S-Bu)_18_]^0^	-		
	[Au_4_Pd_2_(PET)_8_]^0^	-	2017	[143]
	[Au_4_Pt_2_(SCH_2_PhCl)_8_]^0^	-	2020	[142]
	[Au_4_Pt_2_(PET)_8_]^0^	-		
	(AuAg)_34_(A-Adm)_20_	-	2020	[146]
Formation of Ag−O, Ag−S, Ag−Cl bond, etc. (Figure 2B)	Ag_20_(CO_3_)(S-*^t^*Bu)_10_(CH_3_COO)_8_(DMF)_2_	-	2014	[147]
	Ag_18_(CO_3_)(S-*^t^*Bu)_10_(NO_3_)_6_(DMF)_4_	-	2017	[148]
	Ag_44_(V_10_O_28_)(S-Et)_20_(PhSO_3_)_18_(H_2_O)_2_	-	2019	[149]
	Au_7_Ag_8_(dppf)_3_(CF_3_COO)_7_	-	2019	[150]
Control of counter ion (Figure 2C)	[Au_21_(S-*c*-C_6_H_11_)_12_(DPPM)_2_]^+^	[AgCl_2_]^−^	2018	[152]
	[Au_21_(S-*c*-C_6_H_11_)_12_(DPPM)_2_]^+^	[Cl]^−^		
	[Ag_29_(BDT)_12_(PPh_3_)_4_]^3−^	[Cs]^+^	2019	[154]
Introduction of linker molecule (Figure 2D)	Ag_14_(DT-*o*-C)_6_	pyrazine *^a^*	2018	[156]
	Ag_18_(PhPO_3_)(S-*^t^*Bu)_10_(CF_3_COO)_2_(PhPO_3_H)_4_	bpy-NH_2_ *^a^*	2019	[157]
	Ag_15_Cl(S-*^t^*Bu)_8_(CF_3_COO)_5.67_(NO_3_)_0.33_(DMF)_2_	bpy *^a^*	2019	[158]
	Ag_10_(CF_3_COO)_4_(S-*^t^*Bu)_6_(CH_3_CN)_2_	*p*-iah *^a^*	2019	[159]
	Ag_10_(CF_3_COO)_4_(S-*^t^*Bu)_6_(CH_3_CN)	*o*-iah *^a^*		
	Cd_6_Ag_4_(S-Ph)_16_(DMF)_3_(CH_3_OH)	bpe *^a^*	2020	[160]

*a* See Scheme 2.

**Table 2:** Connection modes, NCs, linkers, publication years, and references for 2D CS.

Connection Mode	NC	Linker	Year	Ref.
Formation of Ag−O, Ag−S, Ag−Cl bond, etc. (Figure 2B)	Ag_20_(CO_3_)(S-*i*Pr)_10_(CF_3_COO)_9_(CF_3_COOH)(CH_3_OH)_2_	-	2017	[148]
	Ag_20_(CO_3_)(S-Cy)_10_(CF_3_COO)_10_(CF_3_COOH)_2_(H_2_O)_2_	-		
	Ag_46_(V_10_O_28_)(S-Et)_23_(PhSO_3_)_15_(CO_3_)	-	2019	[149]
	Ag_11_Cl(N-L)_8_(CF_3_COO)_2_·2CHCl_3_	-	2019	[166]
	Ag_11_Cl(N-L)_8_(NO_3_)_2_·2CHCl_3_	-		
	Ag_11_Cl(N-L)_8_(CF_3_SO_3_)_2_·2CHCl_3_	-		
Introduction of linker molecule (Figure 2D)	Ag_12_(S-*^t^*Bu)_6_(CF_3_COO)_6_	bpy *^a^*	2018	[169]
	Ag_14_(DT-*o*-C)_6_	dipyridin-4-yl-diazene *^a^*	2018	[156]
	Ag_12_(S-*^t^*Bu)_6_(CF_3_COO)_6_	TPPA *^a^*	2018	[170]
	Ag_12_(S-*^t^*Bu)_6_(CF_3_COO)_3_	TPyP *^a^*	2019	[171]
	Ag_14_Cl(S-*^t^*Bu)_8_(CF_3_COO)_5_(DMF)	bpy *^a^*	2019	[158]
	Ag_10_(CF_3_COO)_4_(S-*^t^*Bu)_6_(CH_3_CN)_4_	*m*-iah *^a^*	2019	[159]
	Ag_12_(S-*^t^*Bu)_6_(CF_3_COO)_6_(CH_3_CN)_6_	bpz-NH_2_ *^a^*	2019	[172]

*a* See Scheme 2.

**Table 3:** Connection modes, NCs, linkers, publication years, and references for 3D CS.

Connection Mode	NC or Metal Ion	Linker	Year	Ref.
Formation of Ag−O, Ag−S, Ag−Cl bond, etc. (Figure 2B)	Ag_14_(S-*i*Pr)_6_(CF_3_COO)_11_(H_2_O)_3_(CH_3_OH)	-	2017	[148]
	Ag_44_(Mo_6_O_19_)(S-Et)_24_(SCl_4_)_3_	-	2019	[149]
	Ag_17_(C_5_NS_2_H_10_)_14_	-	2019	[173]
Control of counter ion (Figure 2C)	[Au_1_Ag_22_(S-Adm)_12_Cl]^2+^	SbF_6_^−^	2020	[175]
	[Au_1_Ag_22_(S-Adm)_12_]^3+^	SbF_6_^−^		
Introduction of linker molecule (Figure 2D)	Ag_12_(S-*^t^*Bu)_8_(CF_3_COO)_4_	bpy *^a^*	2017	[168]
	Ag_14_(DT-*o*-C)_6_	1,4-bis(4- pyridyl)benzene *^a^*	2018	[156]
	Ag_10_(S-*^t^*Bu)_6_(CF_3_COO)_2_(PhPO_3_H)_2_	bpy *^a^*	2018	[176]
	Ag_12_(S-*^t^*Bu)_6_(CF_3_COO)_6_	CPPP *^a^*	2019	[177]
	Ag_12_(S-*^t^*Bu)_6_(CF_3_COO)_6_ Ag_8_(S-*^t^*Bu)_4_(CF_3_COO)_4_	tppe *^a^*	2019	[178]
	Ag^+^	[C(Au-mdppz)_6_](BF_4_)_2_ *^a^*	2014	[179]

*a* See Scheme 2.
